# Making Carbon-Fiber Reinforced Epoxy-Amine Thermoset Composites More Circular through Chemical Recycling by Catalyzed Solvolysis

**DOI:** 10.1021/acssuschemeng.5c11689

**Published:** 2026-01-29

**Authors:** Valeria De Fabritiis, Leonardo Matta, Gianmarco Griffini, Stefano Turri

**Affiliations:** Department of Chemistry, Materials and Chemical Engineering “Giulio Natta”, 18981 Politecnico di Milano, Piazza Leonardo da Vinci 32, 20133 Milano, Italy

**Keywords:** chemical recycling, solvolysis, Lewis acid, epoxy-amine, carbon-fiber-reinforced composite, end-of-life

## Abstract

The use of carbon-fiber reinforced thermoset polymers (CFRPs) is continuously growing in a wide range of manufacturing sectors, particularly when high performance, lightweight design, and corrosion resistance are required. However, their multimaterial cross-linked structure hinders their recyclability, resulting in the extensive generation of heterogeneous wastes. Nowadays, the correct management of end-of-life (EoL) thermosetting composites remains an open and unsolved issue. In this respect, this work presents a chemical recycling process of a model CFRP from an epoxy-amine network, operated at atmospheric pressure, relatively low temperature (≤200 °C), and mild pH (4–5), allowed by the modification of a Lewis acid catalyst. This process leads to complete liberation of the reinforcing carbon fibers without dimensional alteration, with mechanical characteristics fully comparable to the corresponding virgin fibers, and with the formation of a reusable oligomeric fraction. The recovered components are successfully upcycled by fabricating second-generation CFRPs. Finally, the solvolysis process is validated on real EoL composite parts from aerospace and sports equipment products. This work proposes an economically feasible, safe, and scalable approach to efficiently recycle amine-cured epoxy-based CFRPs, with reusability of all fractions and minimization of any secondary waste generation.

## Graphical abstract

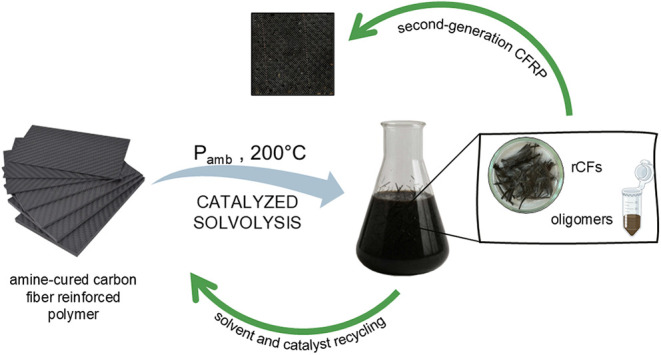

## Introduction

Fiber-reinforced polymers (FRPs) are among the most widely employed materials in many manufacturing sectors. Indeed, thanks to their high strength-to-weight ratio and stiffness, they are extensively used in a large variety of industrial fields where high mechanical, thermal, and chemical responses are required, including energy, constructions, naval, sport, and aerospace sectors. In these fields, the use of thermosets as a polymeric matrix, usually combined with glass or carbon fibers (CFs) as reinforcement, is continuously increasing.^1–4^ The global composites market size was valued at U.S. $100 billion in 2023, with an expected growth of 7.2% per year from 2023 to 2030, and accounted for total volumes around 13 million tons in 2023, with an annual growth rate of approximately 5%.^5–7^ At present, the available recycling processes for FRPs face significant challenges due to the heterogeneity and the irreversible nature of the three-dimensional cross-linked networks characterizing these materials.^8–10^ Currently, common practices for managing composite scraps and end-of-life (EoL) wastes mainly consist in landfilling or incineration,^2–4,10^ which are clearly inconsistent with the principles of the green chemistry and the circular economy. The European ban on wind turbine landfilling by 2025, combined with the European Circular Economy Action Plan,^11,12^ has highlighted the urgent need for developing sustainable recycling solutions for FRPs. Nowadays, three different technologies are employed, namely mechanical, thermal and chemical recycling, but there are still some limitations related to emissions, economic profitability, quality, and possible reusability of the recovered components,^13–16^ limiting potential valorization in second-life applications. The demand for safe and efficient processes, along with the need to produce higher-quality recycled materials, is driving the development of advanced recycling technologies that enable the recovery of both clean, intact fibers and minimally degraded organic matrices.^9,17^ In particular, carbon-fiber reinforced polymers (CFRPs) are ten-times economically more valuable than glass FRPs, driving the monetary value of the global composites market.^1^ Consequently, there is an increasing interest in recycling techniques of these composites, mainly focused on the recovery and reuse of the carbon fibers (CFs) reinforcement. Actually, CFs account for 50–70 wt % of total CFRP material, with an average cost of 20–80 $/kg and even more. Hence, the use of recycled CFs for the fabrication of composites would lead to a significant reduction of the total material production costs, being beneficial from both an environmental and an economic point of view.^1,18^

Amine-cured epoxy resins represent the most commonly used matrix for CFRP fabrication,^19^ but, at the same time, they are particularly recalcitrant to depolymerization due to the robustness of C–N primary bonds. Indeed, for recycling purposes this class of thermosets is usually decomposed under harsh conditions, namely supercritical fluids,^20–25^ strong acids or oxidants,^26–32^ which could damage the integrity of the reinforcing fibers and randomly break the covalent bonds within the matrix macromolecular network, producing organic residues with very limited reusability. One of the most promising chemical recycling technologies that allows for obtaining high-quality CFs and a potentially reusable organic fraction is represented by catalyst-assisted mild solvolysis.^33^ Epoxy-amine thermosets can be depolymerized by the action of Lewis acids as catalysts in different carriers. In this way, the C–N and (partially) C–O bonds can be selectively cleaved, leading to the formation of an organic fraction with residual functional groups and the complete liberation of the fibers, with retained mechanical properties and integrity.^34–37^ In the recent literature, Deng et al. reported ZnCl_2_/H_2_O as the weekly coordinated degradation system in an aqueous medium, but extremely high concentrations of ZnCl_2_ (higher than 60 wt %) were found to be necessary to obtain a complete degradation of the sample.^38^ A similar degradation system was exploited by Hao et al., who employed lower concentrations of ZnCl_2_ (around 20 wt %) in an aqueous buffer, but acetic acid was added to the solution to complete matrix decomposition.^36^ Alternatively, organic solvents can be selected as reaction media. Wang et al. employed AlCl_3_/acetic acid as the reaction system to effectively decompose epoxy-amine CFRP. Indeed, the weekly coordinated Al^3+^ ions selectively cleave the C–N bonds of the resin, while leaving C–C and most of C–O bonds intact.^34^ Similarly, Liu et al. developed an effective ZnCl_2_/ethanol decomposition system, whose efficiency was attributed to the strong swelling ability of ethanol, combined with the strong coordination effect of Zn^2+^ ions on C–N bonds.^17^ In all the reported studies, the use of metal halides as catalysts results in strong acidic conditions (pH ≈ 1 or lower), involving therefore the use of high-cost special alloys for the realization of the chemical plant. Therefore, autoclave pressure reactors are needed to successfully solvolyze the epoxy-amine polymeric matrix, yielding high capital expenses (CapEx) for the realization of the prospective chemical recycling plant as well as safety and scalability limitations.

To overcome these issues, in this work we demonstrate that CFRP based on an epoxy-amine matrix can be efficiently decomposed at atmospheric pressure, relatively mild temperatures (≤200 °C), in organic solutions with moderate pH (pH = 4–5), with the final aim to design a scalable, safer, simple, and cost-competitive recycling process. At the end of the process, the catalytic mixture can be easily recovered by precipitation and filtration and reused for different solvolysis processes. The mild recycling conditions used allow one to obtain recycled CFs that retain >95% of their pristine mechanical properties. In addition, the recovered organic fraction can be successfully reused in the reformulation of fresh epoxy resins.

## Experimental Section

### Materials

Diglycidyl ether of bisphenol A (DGEBA) as epoxy resin and diethylenetriamine (DETA) as polyamine curing agent, iron chloride (FeCl_3_, >98%, anhydrous), zinc chloride (ZnCl_2_, >98%, anhydrous) and aluminum chloride (AlCl_3_, >98%, anhydrous) as Lewis acids, and bis­(2-ethylhexanoato)­hydroxyaluminum were purchased from Merck and used as received. All the solvents, i.e., acetone, tetrahydrofuran (THF), 2-ethylhexanoic acid (2-EHA), and pelargonic acid (PA), were purchased from Merck and used as received, with the exception of 2-EHA, which was magnetically stirred overnight with sodium sulfate (Merck), and then filtered on paper before use. C-200/T fabric used as bidirectional (0/90°) woven CFs (areal density 200 g/m^2^) were purchased from Mates Italiana (Italy) and used as reinforcements. Fragments of EoL tennis rackets were kindly provided by HEAD Sports GmbH, and fragments of EoL aerospace floor panels were kindly supplied by Geven spa.

### Chemical Recycling by Solvolysis

The solvolysis of the epoxy thermoset and of CFRP was performed in a 100 mL round-bottom flask with a water-cooled reflux condenser. Approximately 1 g of sample (around 45 × 10 × 1 mm^3^), the catalytic mixture (composed of 90 wt % of organometallic Al-coordination complex and 10 wt % of AlCl_3_), and the solvent (5–20 wt % with respect to sample weight) were added to the reaction flask. At this point, the system was heated to the reaction temperature and kept under gentle magnetic stirring. The process was conducted under nitrogen at atmospheric pressure and at different temperatures (170–200 °C) and reaction times (6–12 h). At the end, acetone was added to the liquid phase (previously cooled down to room temperature) as nonsolvent for the catalyst, allowing catalyst precipitation and subsequent separation by filtration. Successively, the solvent and the organic fraction were separated from the residual solution through vacuum distillation at 100–120 °C. Considering CFRP solvolysis, the liberated fibers were manually extracted and rinsed with acetone in an ultrasonic bath (30 min) and then dried at room temperature. The degradation ratio (*D*_r_), calculated according to eq 1, permits the evaluation of the extent of dissolution of the polymeric matrix material

1 $$D_{r}=\left(1-\left(m_{1}-m_{0}\cdot w_{\mathrm{fibers}}\right)/\left(m_{0}\cdot w_{\mathrm{resin}}\right)\right)\times 100\%$$

where *m*_0_ represents the weight of the sample before solvolysis, *m*_1_ the final weight, *w*_fibers_ and *w*_resin_ are the weight percentages of CFs and resin (deducted from the thermogravimetric analysis (TGA) analysis on the pristine sample).

## Results and Discussion

### Thermoset and CFRP Characterization

The model thermoset polymer and the corresponding CFRP were first characterized to assess their thermomechanical response in comparison to commonly reported standard epoxy-amine networks (see more in Sections S3 and S4 in the Supporting Information). The Young’s modulus of the thermoset was found to be around 2.5 GPa at 25 °C, with a *T*_g_ = 135 °C, in line with the literature values.^39^ On the other hand, the Young’s modulus of the CFRP (one layer of mat CFs 0/90°) was around 11 GPa.

### Development of the Solvolysis Process: Solvent and Lewis Acid Catalyst Selection

According to recent literature studies,^33–36^ catalyst-assisted solvolysis allows for the effective depolymerization of aliphatic epoxy-amine networks in relatively mild temperature conditions. Among the various options, the use of a Lewis acid in carboxylic acid media appears as one of the most effective strategies. Different from the other cited studies, a key advantage of the solvolysis process proposed in this work is its operation at atmospheric pressure, resulting in several benefits in terms of safety, simplified reactor design, greater scalability, and enhanced energy efficiency. Accordingly, carboxylic acids with a sufficiently high boiling point were selected to design a process temperature higher than the *T*_g_ of the thermoset under study (135 °C, Figure S2 in the Supporting Information), and also promoting the dissolution kinetics of the resin.^32,40^ In addition, the safety of the solvents was taken into account: only low toxicity, potentially bioderivable and (readily or inherently) biodegradable carboxylic acids were included in the solvent design phase (Section S5, Supporting Information), according to the principles of green chemistry.^41–43^ Finally, Hansen solubility theory^44–47^ was followed to provide a selection of the most thermodynamically affine solvents for the epoxy-amine resin swelling and dissolution (see more details in the Section S5, Supporting Information). Following these selection criteria, two different carboxylic acids were identified for further investigations, namely, 2-ethylhexanoic acid (2-EHA) and pelargonic acid (PA). Preliminary solvolysis tests were performed on the thermoset samples to select the solvents and the most effective Lewis acid, i.e., AlCl_3_, FeCl_3_, and ZnCl_2_. Specifically, only metal salts with Cl^–^ as the anion were investigated, due to their higher catalytic effect on the sample degradation process.^9,17,37^ Typically, 10 wt % resin (mass fraction calculated with respect to the solvent weight) was loaded into the reaction flask, 10 wt % Lewis acid (computed with respect to the total weight of the reactants) was added, and the solvolysis reactions were conducted for 6 h at 200 °C. Complete dissolution of the thermoset (calculated according to eq 1) was achieved only when AlCl_3_ was used, while the degradation ratio was 95 and 80% with FeCl_3_ and ZnCl_2_, respectively. Unsurprisingly, the reclaimed organic fraction at the end of the solvolysis process, when metal chlorides/–COOH mixtures were employed as degradation system, featured very high acidity (pH < 1) that would result in difficult scale-up and reutilization of the recyclates.

In addition to ensuring full dissolution of the epoxy-amine thermoset, AlCl_3_ could also be easily precipitated as a solid phase from the organic solution at the end of the reaction. This was not possible when the Zn and Fe salts were used. In order to better investigate the chemical structure and residual catalytic activity of the solid precipitate, an in-depth characterization of this system was performed, as will be discussed in detail. From a chemical standpoint, the pristine AlCl_3_ was found to quantitatively convert into an organometallic coordination complex. The first hypothesized structure of such a complex is shown in Figure 1. In terms of residual catalytic activity of this organometallic complex, as such and in blend with different fractions of neat AlCl_3_, the optimal trade-off between catalytic efficiency, solution pH, and potential reusability of the organic fraction was found when the catalytic mixture was composed by 90 wt % organometallic coordination complex and 10 wt % pure AlCl_3_ (Table S5 in the Supporting Information). In this way, complete degradation of the sample could be achieved, coupled with the formation of an organic recyclate fraction with milder pH (4–5), beneficial both for future reusability of the recyclates and for easier and more economically profitable process scale-up. For these reasons, this 90:10 w/w mixture was selected to be employed as a “modified catalyst” for all the following solvolysis reactions, as will be discussed in detail in the following.

**Figure 1:**
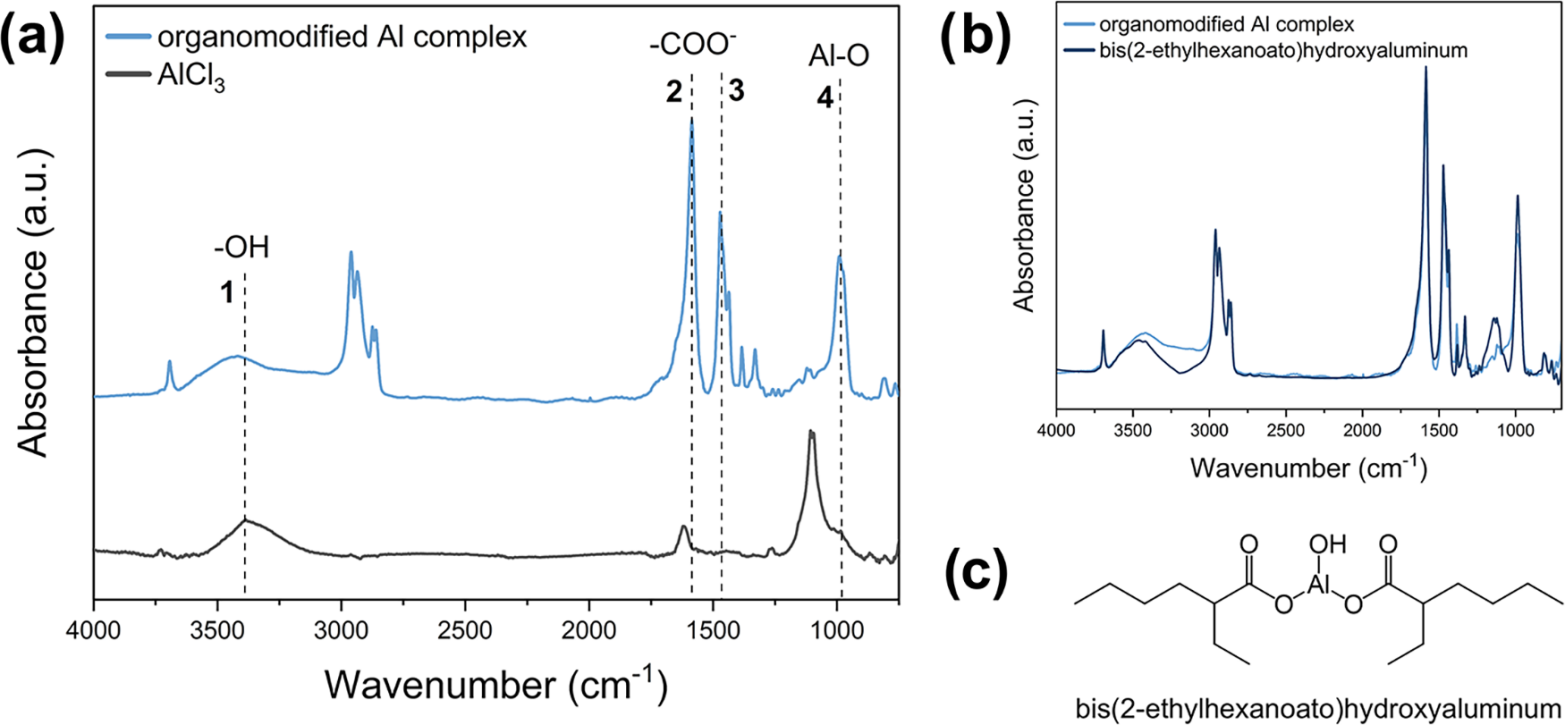
(a) Fourier transform infrared (FTIR) spectra of organometallic coordination complex, recovered after precipitation and filtration at the end of the solvolysis process with 2-EHA as carboxylic acid, and of AlCl_3_; (b) FTIR spectra of organometallic coordination complex, obtained after precipitation and filtration at the end of the solvolysis process with 2-EHA as carboxylic acid, and of commercial bis­(2-ethylhexanoato)­hydroxyaluminum; and (c) molecular structure of bis­(2-ethylhexanoato)­hydroxyaluminum.

### Organometallic Coordination Complex: Catalytic Mixture Characterization and Recycling

The solid precipitate obtained at the end of the solvolysis process with Al^3+^ catalysis was easily separated from the organic mixture by filtration and was characterized. From Figure 1, where the FTIR spectra of the recovered solid are compared with the one of fresh AlCl_3_, it is evident that the solid precipitate is no longer pure AlCl_3_. Instead, the Lewis acid has almost completely transformed into an Al-coordination complex, whose molecular structure will depend on the nature of carboxylic acid employed for the reaction (Section S6 in the Supporting Information), in line with the literature.^34^

Considering the FTIR spectra of the precipitate (Figure 1a), a signal centered at around 3400 cm^–1^ (peak 1, Figure 1a) can be attributed to the −OH stretching, and the narrow and strong bands at 1582 and 1469 cm^–1^ (peaks 2 and 3 of Figure 1a, respectively) are the asymmetric and symmetric stretching vibrations of COO^–^. As reported in the literature,^48–50^ the band at around 980 cm^–1^ (peak 4, Figure 1a) is due to the stretching vibration of Al–O. To confirm this hypothesis, the FTIR spectra of the organometallic coordination complex (obtained employing AlCl_3_/2-EHA as solvolytic degradation system) was compared with the one of commercial bis­(2-ethylhexanoato)­hydroxyaluminum, as reported in Figure 1b. Based on the excellent match between the two FTIR spectra, it can be concluded that the recovered solid at the end of the solvolysis process is mainly composed of the bis­(2-ethylhexanoato)­hydroxyaluminum complex, which results from the degradation reaction. This evidence is further corroborated by electrospray ionization mass spectrometry (ESI-MS) and elemental analysis on Al complexes formed when using PA and 2-EHA (Section S6 in the Supporting Information).

As mentioned, Al^3+^ effectively catalyzes the solvolysis reaction, especially when coupled with Cl^–^. However, the resulting pH of the recovered organic solution after removal of the solid precipitate was found to be lower than 1, mainly due to the generation of HCl in the solvent medium. In order to promote the reusability of the organic recyclate and for an easier and cost-effective future scale-up of this technology, an increased pH for the reaction solution should be sought. For this reason, neat AlCl_3_ was combined with different weight percentages of the organometallic coordination complex recovered after the solvolysis, in the attempt to reach an optimized balance between catalytic activity (*D*_r_), catalyst reusability, and solution pH. In particular, the resulting *D*_r_ and solution pH were found to vary for different catalytic systems by varying proportions of recovered organometallic Al-coordination complex and pristine AlCl_3_ (see Table S5 in the Supporting Information). Interestingly, when the catalytic mixture was composed of 90 wt % organometallic coordination complex and 10 wt % AlCl_3_, a complete dissolution of the thermoset could be achieved in the given process conditions (200 °C, 8 h), in the presence of a milder pH solution (pH = 4–5). In addition, in line with green chemistry and circular economy principles, it was verified that the catalytic system recovered after the reaction could be successfully reused at least three times for further solvolysis processes, by proper coformulation with 10 wt % of fresh AlCl_3_. These evidence suggest that largely reduced costs of industrial production for the catalytic system can be envisaged in future upscaling scenarios through its recovery and reuse, with no secondary waste generated during the entire process.

### Mild Solvolysis Process of Thermoset and CFRP

Based on the encouraging results obtained on *D*_r_ and on the pH of the reaction solution by reformulating the catalytic system (90 wt % recovered organometallic Al-complex, 10 wt % fresh AlCl_3_), a more in-depth study was performed to elucidate the effects on the efficiency of the solvolysis process (*D*_r_) of different process parameters, namely epoxy resin concentration, modified-catalyst concentration, reaction time and reaction temperature (Table S6 in the Supporting Information). As highlighted in a previous study, all the variables showed an influence on the *D*_r_ of the sample.^33^ From a perspective of potential industrial exploitation of the process, the concentration of the material to be recycled should be maximized, while the concentration of the catalyst, the reaction time, and the reaction temperature should be minimized.^34,36^ Specifically, a lower concentration of the thermoset would be meaningless from a scale-up perspective, while excessively high values hinder the complete degradation of the sample, reducing process efficiency (see line 6 in Table S6, Supporting Information). On the other hand, catalyst concentration must be minimized, while avoiding excessive reaction times that would compromise process efficiency. Finally, lower reaction time and temperature, desirable from an industrial perspective, would prevent complete dissolution of the sample. These factors drove the selection toward two different operating conditions, which were found to yield maximum *D*_r_ while ensuring minimized catalyst use and reaction time. In particular, with an epoxy resin concentration of 10 wt %, reaction times of 6 or 8 h would ensure complete sample dissolution (*D*_r_ > 99%) in the presence of 10 or 5 wt % concentration of modified catalyst, respectively (line 2 and 4 in Table S6, respectively).

These operating conditions were also tested and validated on the corresponding CFRP materials. However, considering a fiber content in the composite material of approximately 50 wt % (TGA analysis reported in Figure S5, Supporting Information), the resulting CFRP concentration in the reaction solution was set to 25 wt %, corresponding to a polymeric matrix content of 12.5 wt %. Complete dissolution of the resin could be achieved with a solvolysis process carried out at atmospheric pressure, *T* = 200 °C, moderate pH (4–5), with 25 wt % CFRP concentration in the solvent medium and with 5–10 wt % modified-catalyst concentration, in a time frame of 6–12 h (Figure S12 in the Supporting Information). At the end of the process, the CFs could be easily reclaimed from the reaction solution, a residual organic recyclate could be isolated, and the catalytic system could be recovered. Interestingly, this solvolysis process was successfully validated on different epoxy-amine networks (including cycloaliphatic and aromatic polyamines as curing agents—Section S10 in the Supporting Information), demonstrating the wide applicability of such chemical recycling methodology.

### Characterization of the Organic Fraction

At the end of the solvolysis process and of the workup procedure, the organic fraction could be recovered. In particular, the solvent was removed by vacuum distillation, and the residual organic recyclate was characterized. To that end, FTIR spectroscopy (Figure 2a) allowed for the comparison of the chemical structures of the polymeric matrix before (cross-linked epoxy resin with no CF reinforcement) and after (recovered organic fraction) solvolysis.

**Figure 2:**
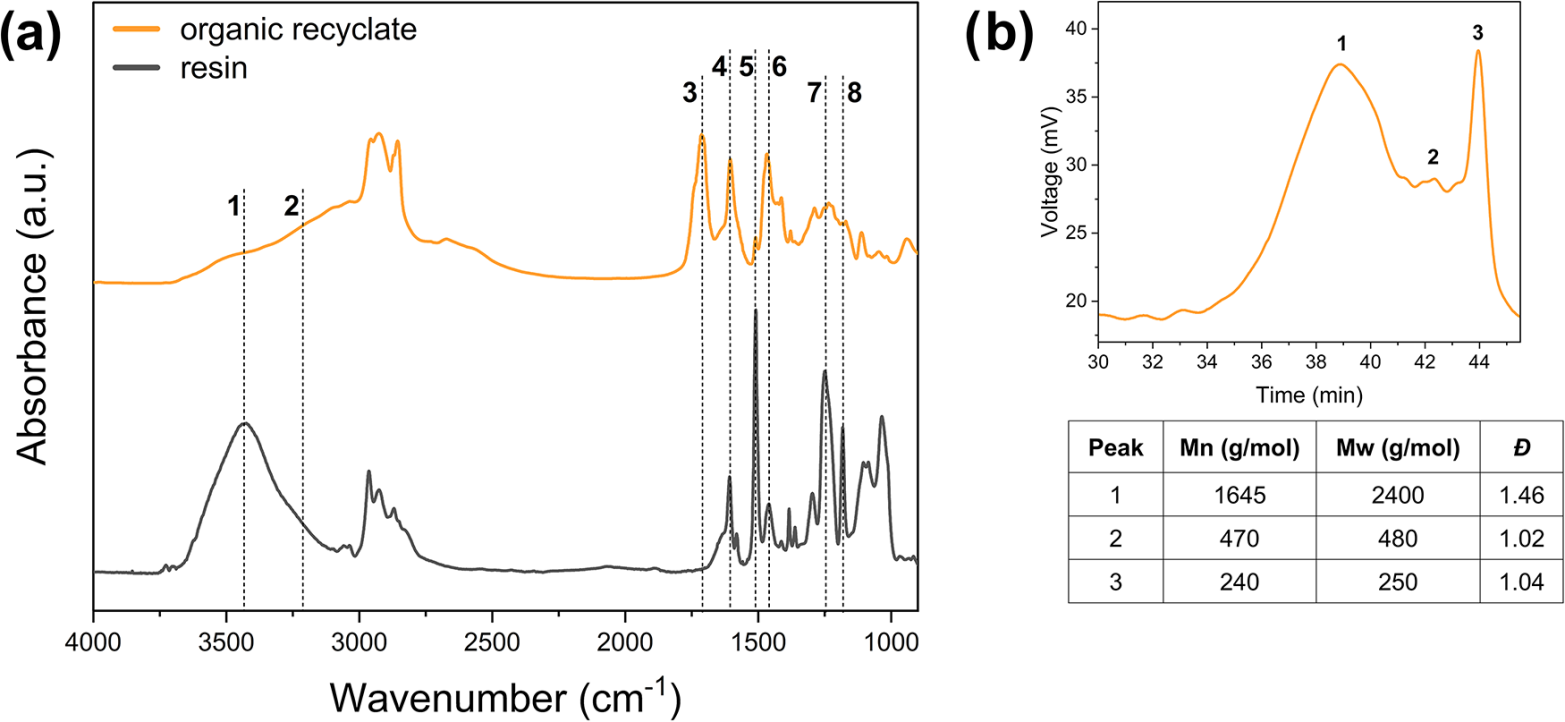
(a) FTIR spectra of pristine resin and organic recyclate and (b) gel permeation chromatography (GPC) analysis of the organic recyclate.

Compared with the FTIR spectrum of the pristine thermoset resin, the organic recyclate showed some distinctive features in the signal centered at around 3300 cm^–1^ (Figure 2a), attributable to stretching vibrations of −OH and –NH groups. Specifically, the intensity of the signal associated with vibrations of −OH groups (∼3400 cm^–1^, peak 1 in Figure 2a) was found to significantly decrease in the FTIR spectrum of the organic product compared with the same signal in the spectrum of the pristine resin, suggesting the presence of esterified chemical structures inside the organic fraction. This conclusion was confirmed by the appearance of a peak at around 1735 cm^–1^, characteristic of C=O stretching vibrations in ester groups (peak 3, Figure 2a). In addition, the appearance of a broad and intense band centered at ∼3200 cm^–1^ (peak 2, Figure 2a), attributable to –NH stretching, likely indicates the presence of amine structures in the organic recyclate, which were not found in the spectrum of the pristine material. The bands at 1600, 1510, and 1460 cm^–1^ (peaks 4, 5, 6 in Figure 2a), typically associated with different C=C vibrations in the aromatic skeleton, indicate the presence of residual intact aromatic rings also in the organic fraction, but with lower concentration with respect to what observed in the pristine resin. The decreased intensity of the signals at 1246 and 1183 cm^–1^ in the FTIR spectrum of the organic recyclate, attributable to C–O and C–N stretching vibrations (peaks 7 and 8 in Figure 2a, respectively), suggests efficient and selective cleavage of these two chemical bonds during the solvolysis reaction. Interestingly, the selective C–N cleavage was also confirmed by heteronuclear single-quantum coherence (HSQC) NMR analysis (Figure S13, Supporting Information), where the presence of protons not coupled with carbon atoms was reported, confirming the presence of DETA-derived structures in the organic fraction.

The molecular weight and molecular weight distribution of the organic recyclates were evaluated by means of a GPC analysis (Figure 2b), where at least three different peaks can be distinguished. Among them, the peak appearing at the higher retention time (peak 3, 43.9 min) could be hypothetically associated with a bisphenol A derived structure (228 g/mol). Hence, peaks 1 and 2 (37.4 and 28.9 min, respectively) could be related to oligomeric structures derived from the solvolysis reaction, exhibiting higher molecular weights.

Based on the previous results, a potential chemical degradation mechanism taking place during the solvolysis process can be supposed (Figure 3). By the combined action of the carboxylic acid (PA or 2-EHA) and of the catalytic system, selective cleavage of C–N and (partially) C–O bonds can be expected. This may lead to the formation of different DGEBA-derived aromatic species (structures 1 and 2 in Figure 3) and amine structures (structure 3 in Figure 3). The presence of these components in the organic recyclate was also confirmed by NMR spectra (^1^H and ^13^C, Figures S14 and S15 in the Supporting Information) and by ESI-MS (Figure S16 in the Supporting Information).

**Figure 3:**
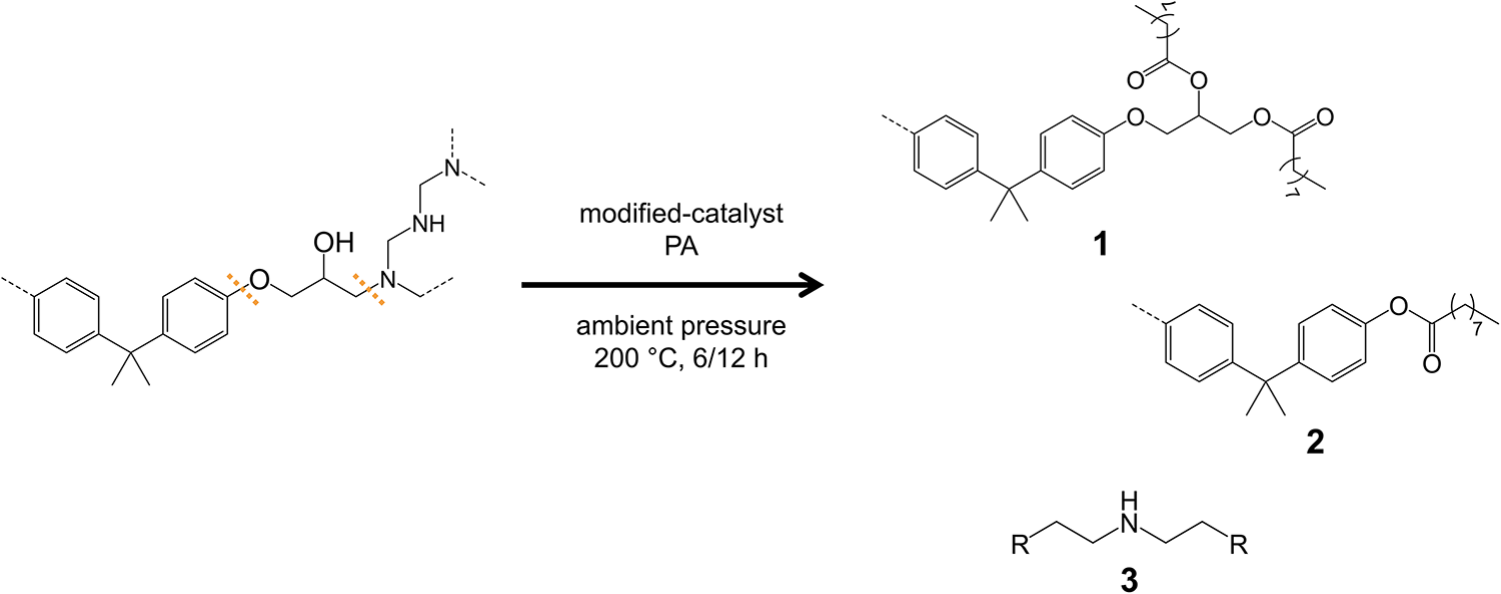
Hypothesized residual oligomeric structures in the organic recyclate after the solvolysis process of the thermoset, employing PA as the reaction solvent.

These pieces of evidence imply that the organic recyclate contains oligomers with residual functional groups, suggesting its potential closed-loop reusability for second-generation composite applications.

### Characterization of the Recovered CFs

The cleanliness and integrity of reclaimed CFs (rCFs) recovered after the solvolysis of CFRPs was assessed through scanning electron microscopy/energy-dispersive X-ray (SEM/EDX) and TGA analyses. SEM images (Figure 4a,b) confirmed that no evident polymer residues were present on the fiber surface, highlighting their preserved morphology after solvolysis treatment. This was further supported by EDX analysis, with the presence of the signal associated with carbon being prevalent over that associated with oxygen potentially originating from the presence of oxide-layers on the fiber surface (Figure S19 in the Supporting Information). However, the presence of a peak correlated with Al may indicate that some residual catalyst particles were still present on the fiber surface.

**Figure 4:**
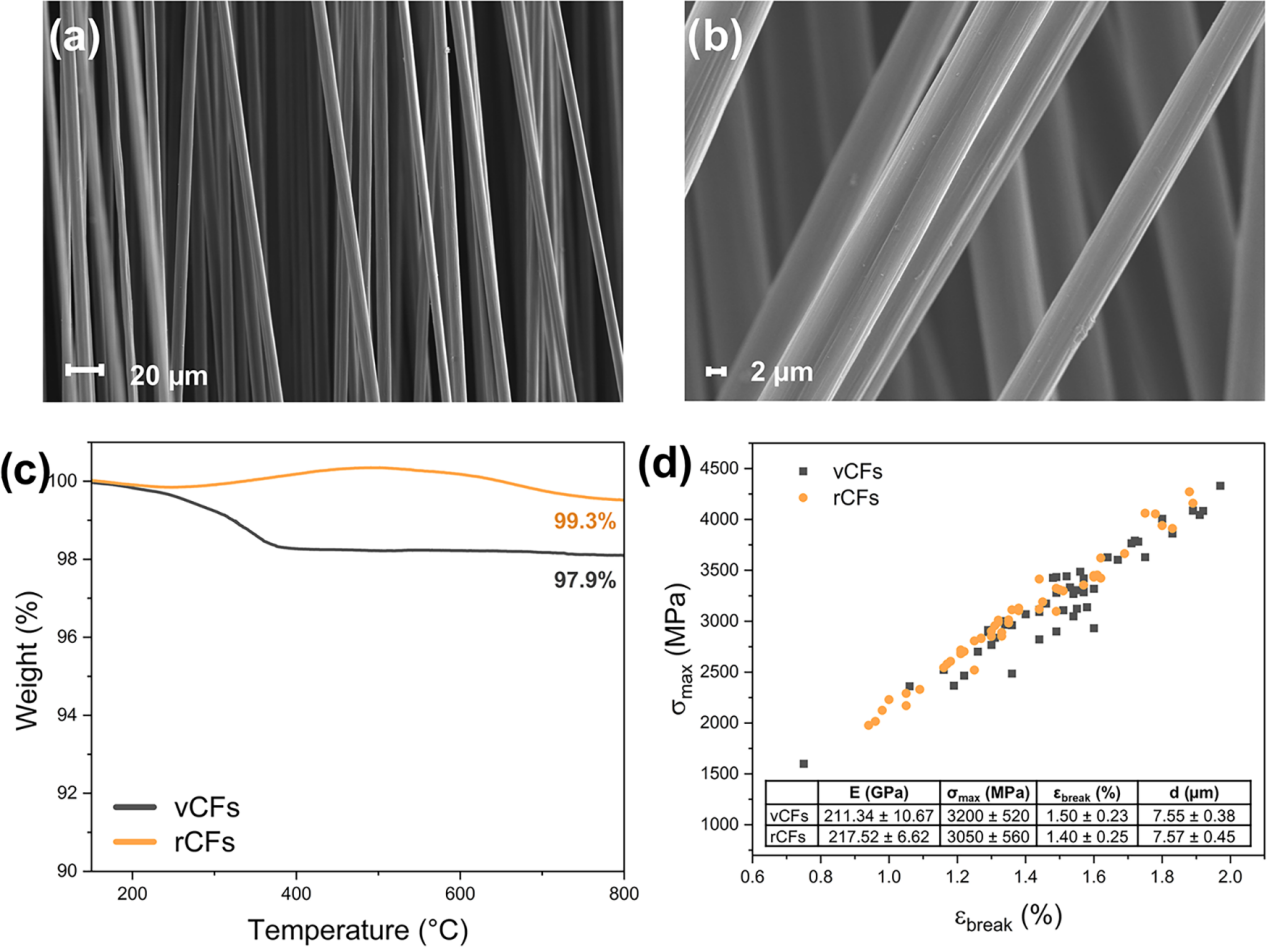
(a) SEM image of virgin CFs (vCFs) at 1.00× magnification; (b) SEM image of rCFs at 5.00k× magnification; (c) TGA thermograms in nitrogen atmosphere of virgin and recovered CFs; (d) maximum stress and maximum strain distribution, for virgin and recycled CFs. Elastic modulus (*E*), stress, and elongation at break (σ_max_ and ε_break_, respectively), and fiber diameter (d) of virgin and recycled CFs are reported in the table.

In line with SEM analysis, TGA measurements of the rCFs confirmed complete removal of the polymeric matrix (Figure 4c). Indeed, the TGA curves of virgin and rCFs appear very similar, with the only exception of a ∼2% mass loss event at around 250–350 °C recorded for the virgin CFs (vCFs), probably related to the volatilization of sizing agents, which are no longer present in rCFs. The mechanical properties of rCFs were also investigated through micromechanical tensile testing, and compared with those of vCFs, to assess their reusability for further applications. Figure 4d shows the tensile test results for all the tested fibers and the resulting average properties (namely, elastic modulus *E*, maximum stress σ_max_, elongation at break ε_break_, and fiber diameter *d*). From the microtensile tests, it is evident that the mechanical properties of rCFs are similar to those of their pristine counterpart. In particular, the values of the elastic modulus and the diameter of rCFs are comparable with the values of vCFs, while the retention of stress and elongation at break are >95 and >93%, respectively. These results confirm that the solvolysis process proposed here allows the recovery of high-quality and potentially reusable rCFs, without affecting their morphological and mechanical characteristics.

### Second-Generation CFRP

The potential circularity of this process was investigated by manufacturing a new CFRP composite material with CFs (both virgin and recycled) and with a fraction of organic recyclate in the reformulated epoxy resin. As previously discussed, the recovered organic recyclate contains oligomers and residual functional groups, mainly amines, esters, and carbonyl groups. Therefore, it was introduced as an extender or reactive diluent in the formulation of fresh DETA-epoxy resin. The effect of the introduction of different amounts of organic recyclate in the resin formulation was evaluated through gel content measurements and differential scanning calorimetry (DSC) analysis. The compositions incorporating 5 and 10 wt % of organic recyclate were found to exhibit similar values of *T*_g_ (∼120 °C, Figure S25 in the Supporting Information), which were comparable with that of the neat DGEBA-DETA thermoset. On the contrary, a slight decrease in *T*_g_ was detected when 20 wt % of the recyclate was included in the resin formulation (∼95 °C, Figure S25 in the Supporting Information). For this reason, 10 wt % of the oligomeric fraction was identified as the maximum content of organic recyclate suitable for epoxy matrix reformulation. Accordingly, second-life CFRPs were manufactured using this modified formulation and virgin and reclaimed CFs from the solvolysis process. Despite the removal of the sizing agent from the CFs during the recycling process,^51–53^ SEM analysis confirmed that excellent impregnation of rCFs could be attained when using the reformulated second-generation epoxy resin matrix material (Figure 5a,b), similarly to the case of pristine reinforcement (Figure 5c). Finally, the mechanical properties of the second-generation CFRPs (Figures 5d and S29 in the Supporting Information, with virgin and reclaimed CFs used as reinforcements, respectively) were compared to those of virgin CFRPs (shown in Figure S7).

**Figure 5:**
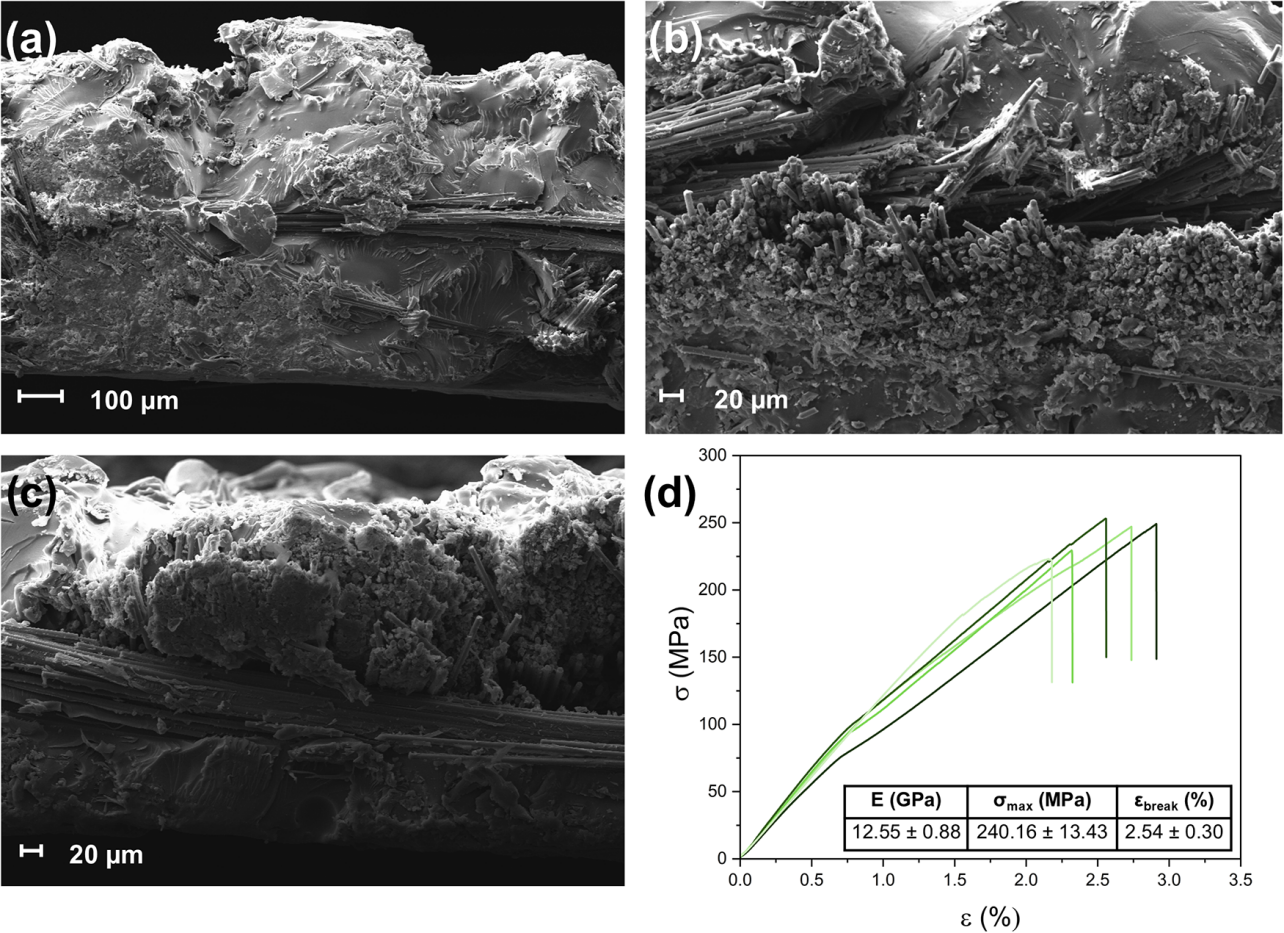
(a, b) SEM images of two sections of second-generation CFRP, with rCFs and 10 wt % oligomeric fraction, at 250× and 500× magnification; (c) SEM image of second-generation CFRP, with vCFs and 10 wt % oligomeric fraction, at 500× magnification; and (d) stress–strain curves of second-generation CFRP specimens with one layer of mat vCFs (0/90°) and DGEBA-DETA network incorporating 10 wt % of organic recyclates. The table shows the results of tensile testing in terms of elastic modulus (*E*), maximum stress (σ_max_), and elongation at break (ε_break_). Stress–strain curves of second-generation CFRP specimens with one layer of rCFs mat and DGEBA-DETA network with a 10 wt % oligomeric fraction are reported in Figure S29 in the Supporting Information.

Interestingly, second-generation CFRPs incorporating vCFs (Figure 5d) were found to exhibit mechanical properties in line with those shown by virgin CFRPs (Figure S7 in the Supporting Information), confirming the straightforward reusability of the 10 wt % oligomeric fraction as an extender for the preparation of the epoxy polymeric matrix. On the other hand, the use of rCFs for the fabrication of second-generation CFRPs (Figure S29 in the Supporting Information) yielded a significant reduction of *E* and σ_max_ compared with the virgin counterpart (−33.3 and −54.1%, respectively). Considering that the physical properties of rCFs were demonstrated to be well preserved during the chemical recycling process, this effect may be related to the misalignment of the fibers in the rCF mat after the solvolysis (clearly shown in Figure S12 in the Supporting Information), resulting in a less effective load distribution in the resulting second-generation CFRP.

### Real EoL Components: Sports Equipment and Aerospace Sectors

The suitability of the reported operating conditions was validated on real EoL CFRP parts made of different epoxy-amine networks. The sports equipment and the aerospace sectors extensively use this type of composite due to their high strength characteristics, their excellent chemical resistance, and their lightweight. Accordingly, fragments of tennis rackets and of sandwich panels from structural parts in passenger aircraft (see Section S12 in the Supporting Information for more characteristics) were selected and subjected to the solvolysis treatment, exploiting the mild reaction conditions discussed in the previous sections and validated on model CFRP. Interestingly, complete liberation of the reinforcing fibers could be achieved in both cases (Figure 6), demonstrating the practical relevance of the mild recycling process proposed in this work and its straightforward applicability in the EoL management of such compounds.

**Figure 6:**
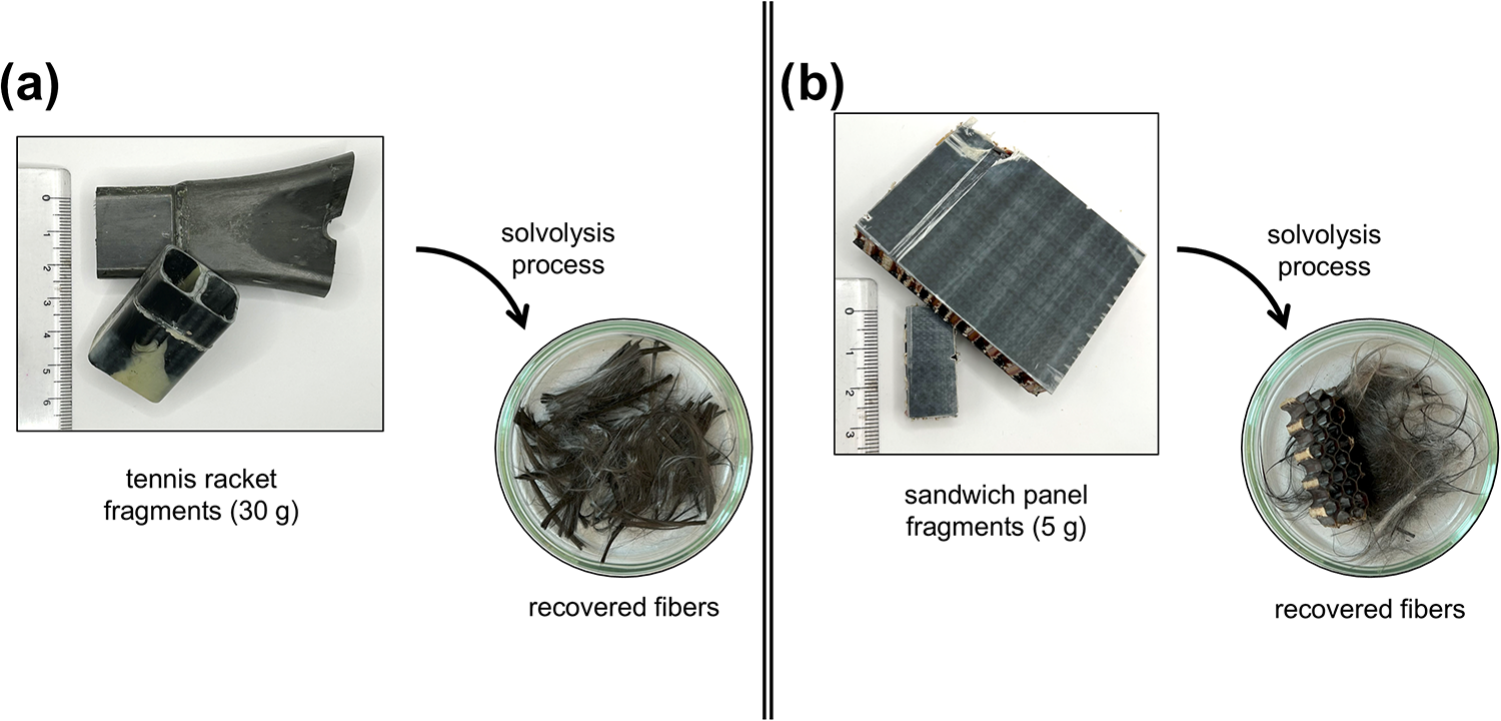
Solvolysis treatment of end-of-life components: (a) tennis racket fragments and (b) sandwich panel fragments. The solvolysis was carried out using the same reaction conditions reported for model epoxy systems, namely, 40 wt % EoL fragment, 10 wt % modified catalyst, 200 °C, *P*_amb_, 8 h.

### Material Circularity Assessment

The circularity potential of the chemical recycling process proposed in this work could be preliminarily quantified by focusing on two key elements of the process, namely the reaction solvent and the catalyst, and by calculating their corresponding material circularity indicator (MCI), as originally proposed by the Ellen MacArthur Foundation.^53–56^ To that end, in line with the results presented here, it was assumed that both the catalyst and the reaction solvents could be recovered with a recycling efficiency of at least 90% and reused for multiple solvolysis cycles, contributing to a significant reduction in virgin feedstock (viz., solvent and catalyst) requirements and thus potentially enhancing the overall circularity of the process.

Furthermore, an additional assessment of the potential impact of this solvolysis process on the circularity of CFRPs was also carried out, by calculating the MCI for second-generation CFRP materials incorporating rCFs reclaimed from the solvolysis process as only reinforcement and a fraction (10 wt %) of the organic recyclate recovered from the chemical recycling process in the epoxy resin formulation used for the polymer matrix. Indeed, as demonstrated through the fabrication of second-generation CFRPs, the rCFs reclaimed from the solvolysis process were found to be clean, intact, and with >95% retention of their mechanical properties. Similarly, 10 wt % of the organic recyclate could be used as an extender in the formulation of fresh epoxy resin, without detrimental effects on the performance of resulting CFRP materials.

Based on a comparative assessment with a fully linear base-case situation in which only virgin feedstock (solvent and catalyst) is used for the solvolysis process (MCI = 0.1, Section S13 in the Supporting Information), the scenario characteristic of the chemical recycling process presented in this work displayed a significantly higher MCI for both solvent and catalyst (from 0.1 to 0.74 and 0.91 for solvent and catalyst, respectively).^57^ Similarly, the case of second-generation CFRPs fabricated and characterized in this work (featuring only rCFs obtained from the solvolysis process and a resin formulation incorporating 90 wt % of virgin epoxy and 10 wt % of recycled oligomers isolated at the end of the solvolysis process) yielded a substantially higher MCI (0.80) with respect to a reference CFRP (MCI = 0.10) composed only of virgin feedstock (epoxy matrix and CFs).

Such increased MCI values highlight the substantial potential benefits that the solvolysis process proposed in this work can bring about in terms of both reduced consumption of virgin materials and a lower impact associated with the production and disposal of CFRP. Accordingly, the proposed approach may represent a valuable strategy to enhance the circularity of these materials, in line with the principles of the circular economy.

### Conclusive Remarks and Process Scalability

In this work, an efficient solvolysis process for different classes of epoxy-amine-cured CFRP has been demonstrated, showing its flexibility and general applicability in recycling real EoL materials. The process was designed in line with the principles of green chemistry and circular economy and can be carried out at atmospheric pressure, relatively mild temperature (≤200 °C) and moderate pH (4–5), using low-cost, modified Lewis acid catalysts and (preferentially) biodegradable and bioderived organic solvents. These features are relevant to reducing capital costs for the realization of a potential future large-scale recycling plant based on this technology, which is one of the currently known intrinsic limitations of chemical recycling strategies. According to consolidated scale-up rules for chemical plants,^58^ the capital costs for a new process design can be presented in the form of a power law of capacity (eq 2)­

2 $$C_{E}=C_{B}\cdot {\left(Q/Q_{B}\right)}^{M}\cdot f_{M}\cdot f_{P}\cdot f_{T}$$

where *C*_E_ is the cost of the equipment with capacity *Q*, *C*_B_ is the base cost for the equipment of capacity *Q*_B_, *M* is a constant depending on the equipment type, and *f*_M_, *f*_P_, and *f*_T_ are correction factors taking into account the materials of construction of the reactor, the design pressure, and design temperature, respectively. It is interesting to consider how these parameters can change as a function of the process variables. For example, exponent *M* is typically 0.45 for an agitated reactor but increases up to 0.82 for pressure vessels. On the other hand, *f*_M_ is typically 1 for reactors made in standard carbon steel, but it increases up to 2.4 for stainless steel, 3.6 for Hastelloy, and 4.4 for Inconel, which are special alloys resistant to very harsh conditions. Some simple calculations based on these general considerations lead to the conclusion that a process carried out at ambient pressure, low temperature, and moderate pH can dramatically reduce (up to 4–5 times) the cost for the installation of a high-capacity plant.

The results reported here may represent a significant step forward toward a more efficient circular economy of fiber-reinforced composites.

## Supplementary Material



## Notes

Amine-cured epoxy resin characterization, CFRP characterization, with the DGEBA-DETA polymeric matrix, solvent and Lewis acid selection, organometallic coordination complexes characterizations, characterization of the organic fraction, characterization of the recovered CFs, process validation: solvolysis process on different model epoxy resins, second-generation CFRP, calculation of material circularity indicator (MCI), and end-of-life component characterization (PDF)
