# Supplementary material for: Making Carbon-Fiber Reinforced Epoxy-Amine Thermoset Composites More Circular through Chemical Recycling by Catalyzed Solvolysis
Source: ACS Sustain Chem Eng. 2026 Jan 29;14(5):2546–55. doi: 10.1021/acssuschemeng.5c11689 (PMC12892873; doi:10.1021/acssuschemeng.5c11689)
Supplement: Supplementary file 1 [file sc5c11689_si_001.pdf]

Supporting Information

Making carbon-fiber reinforced epoxy-amine thermoset composites more circular through chemical recycling by catalyzed solvolysis

Valeria De Fabritiis[a], Leonardo Matta[a], Gianmarco Griffini[a,*], Stefano Turri[a,*]

[a] Department of Chemistry, Materials and Chemical Engineering "Giulio Natta", Politecnico di Milano, Piazza Leonardo da Vinci 32, 20133 - Milano, Italy

35 pages, 36 Figures, 12 Tables

Corresponding authors: gianmarco.griffini@polimi.it; stefano.turri@polimi.it

Table of contents

S1. Materials S3
S2. Characterization techniques S4
S3. Amine-cured epoxy resins characterization S5
S4. CFRP characterization, with DGEBA-DETA polymeric matrix S7
S5. Solvent and Lewis acid selection S9
S6. Organometallic coordination complexes characterizations S11
S7. Mild solvolysis process of thermoset and CFRP S13
S8. Characterization of the organic fraction S14
S9. Characterization of the recovered CFs S18
S10. Process validation: solvolysis process on different model epoxy resins S19
S11. Second-generation CFRP S22
S12. End-of-life components characterization S25
S13. Calculation of material circularity indicators (MCI) S27
Bibliography S35

S1. Materials

Diglycidyl ether of bisphenol A (DGEBA) as epoxy resin and diethylenetriamine (DETA) as polyamine curing agent, iron chloride ($FeCl_3$, > 98%, anhydrous), zinc chloride ($ZnCl_2$, > 98%, anhydrous) and aluminum chloride ($AlCl_3$, > 98%, anhydrous) as Lewis acids, and bis(2-ethylhexanoato)hydroxyaluminum were purchased from Merck and used as received. All the solvents, i.e. acetone, tetrahydrofuran (THF), 2-ethylhexanoic acid (2-EHA) and pelargonic acid (PA), were purchased from Merck and used as received, with the exception of 2-EHA, which was magnetically stirred overnight with sodium sulfate (Merck), and then filtered on paper before use. C-200/T fabric used as bi-directional (0°/90°) woven CFs (areal density 200 g/m^2) were purchased from Mates Italiana (Italy) and used as reinforcements. Fragments of EoL tennis rackets were kindly provided by HEAD Sports GmbH, and fragments of EoL aerospace floor panels were kindly supplied by Geven spa.

S2. Characterization techniques

Differential scanning calorimetry (DSC) analyses were conducted with a Mettler-Toledo DSC/823e under nitrogen atmosphere. Thermal cycle: from 25 °C to 250 °C at 10 – 20 °C min^{-1}. The curing heat and the glass transition temperature (Tg) were determined from the thermograms as integral of the exothermic signal in the first heating run and as inflection point temperature in the second heating run, respectively. The effectiveness of the crosslinking reaction was checked through gel content measurements by soaking a solid sample (m_{solid} ~ 1 g) in THF (50 mL) under continuous magnetic stirring (24 h, ambient temperature). The sample was dried to completely remove the solvent (60 °C, 24 h), and then weighed (m_2). The gel content percentage was calculated:

$$gel\ content = \frac{m_2}{m_{solid}} \cdot 100\ [\%]$$

Thermogravimetric analysis (TGA), under nitrogen atmosphere, was employed to analyze the fiber fraction of the CFRP, and to assess the cleanliness of recycled CFs (TA Instruments TGA 550). The samples were heated from 25 °C to 900 °C at 10 °C min^{-1}. Fourier-transform infrared (FTIR) spectra, performed on organic fractions and solid residues (through deposition on KBr windows), were collected with a Nicolet iS50 FTIR Spectrometer (Thermo Fisher Scientific). The samples were analyzed in transmission mode (4000 – 600 cm^{-1}), with 4 cm^{-1} resolution and 64 scans.

Gel permeation chromatography (GPC) analysis was conducted at 35 °C with Waters 510 HPLC system, using THF as eluent and polystyrene calibration columns (Waters 2410 refractive index detector). The sample (volume 400 µL, 4 mg/mL in THF) was injected into a system of columns connected in series (Ultrastyragel models HR5, HR4, HR3 and HR2), the analysis was carried out at 1 mL/min. ^{1}H, ^{13}C and HSQC nuclear magnetic resonance (NMR) spectra were recorded on a Bruker Avance II 400 MHz in $CDCl_3$ solution. Scanning electron microscopy (SEM) imaging (coupled with Energy Dispersive X-ray spectroscopy - EDX analysis) was employed to evaluate the composites impregnation and the cleanliness and integrity of recycled CFs. Carl Zeiss AG Evo 50 microscope, operating under high vacuum and using an electron high tension (EHT) of 20 kV and a probe current of 100 pA at 100x and 2000x magnification was employed. Elemental analysis was carried out with CHNS FlashSmart™ (Thermofisher scientific) instrument to determine the content of C, N, H of products. Electrospray ionization mass spectrometry (ESI-MS) analysis was done with a LCQ Fleet Ion Trap LC/MS instrument. Tensile properties were measured with Zwick/Roell BT-FR010TH.A50 and INSTRON 5900R 4505 dynamometers, according to ISO 527-4. The mechanical properties of virgin and recycled CFs (single fiber testing) were evaluated through Textechno FAVIMAT+ instrument (ASTM D 1577). For each sample, 50 fibers were analyzed. The gauge length (L_0) was set to 25 mm, the test speed was 2 mm min^{-1}, the pre-tensions were 1 cN tex^{-1} for linear density and 0.5 cN tex^{-1} for tensile tests measurements. The load cell was 210 cN. The elastic modulus (E, evaluated at 0.5 – 0.6% strain) and the diameter of the fibers were automatically determined by the instrument through a vibroscopic method.

S3. Amine-cured epoxy resins characterization

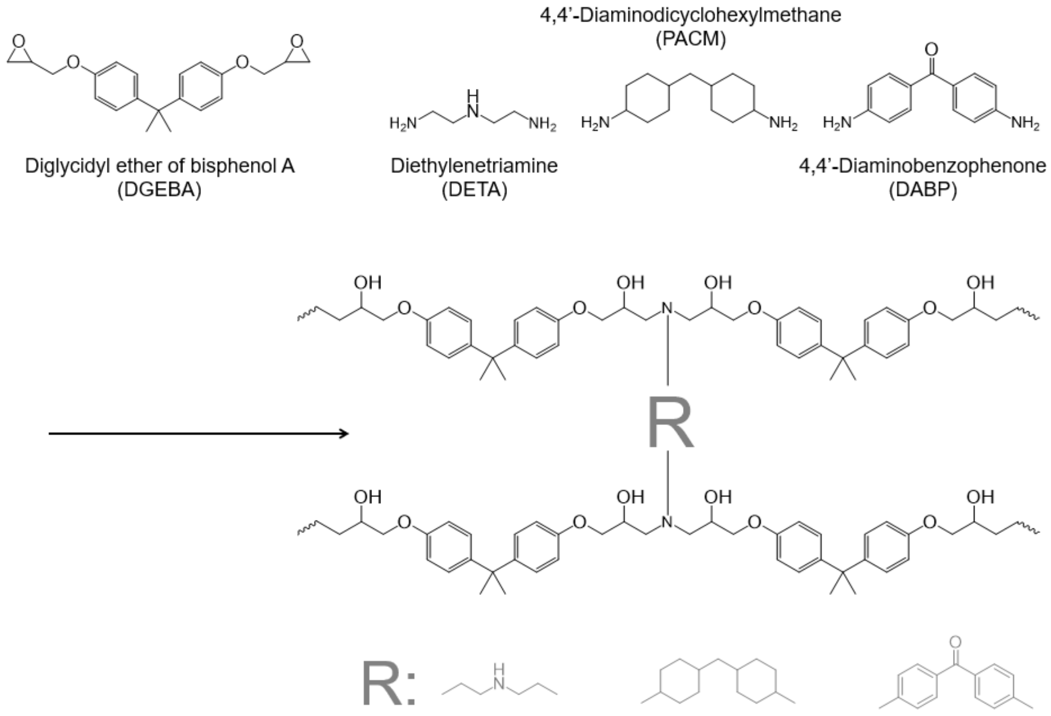

Figure S 1: Schematic reaction mechanism between DGEBA and aliphatic (DETA), cycloaliphatic (PACM) and aromatic (DABP) amine curing agents, and representative structure of the resulting epoxy-amine resins.

The crosslinked epoxy-resins were obtained by mixing DGEBA and the amine curing agent (epoxy:NH 1:1) at room temperature (DETA, PACM) or 120 °C (DABP) for 10 min, obtaining an homogeneous liquid phase. The mixture is poured in silicon molds and cured in ventilated oven according to the curing conditions reported in Table S1, obtaining Tg = 135 °C for DGEBA-DETA, Tg = 180 °C for DGEBA-PACM and Tg = 157 °C for DGEBA-DABP, respectively (DSC curved are shown in Figure S2).
The gel content results > 99% in all the model epoxy systems.

Table S 1: Curing cycles of the epoxy-amine thermosets under investigation.

	**Curing conditions**
DGEBA – DETA	60 °C, 2 h + 120 °C, 2 h + 140 °C, 1 h
DGEBA – PACM	80 °C, 2 h + 130 °C, 1 h + 150 °C, 1 h
DGEBA – DABP	130 °C, 1 h + 180 °C, 2 h + 200 °C, 2 h

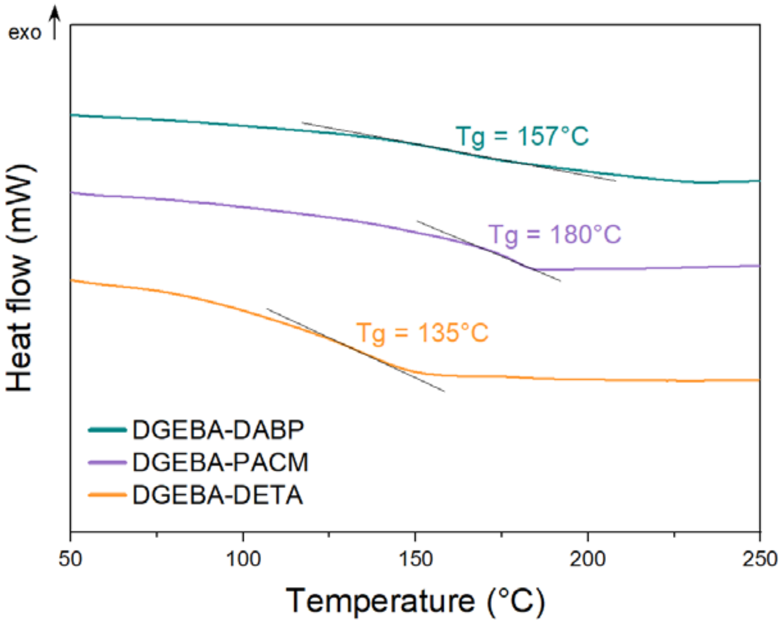

Figure S 2: DSC curves of the epoxy-amine samples with identification of the glass transition temperature (Tg) as inflection point in the second heating run of the thermographs.

Tensile tests were performed on DGEBA-DETA thermoset using a Zwick/Roell BT-FR010TH.A50 dynamometer equipped with a 10 kN load cell, applying a displacement rate of 1 mm min^{-1} at 25 °C, to determine the mechanical properties. The deformation was detected through a long-stroke extensometer. Five rectangular shaped samples with a gauge length of 50 mm, 10 mm width, and thickness of 2 mm were tested. The elastic modulus (E), determined from the slope of the curve between 0.05% and 0.25% of the total strain, strength and elongation at break (σ_{max} and ε_{break}) were evaluated and reported in Figure S3:

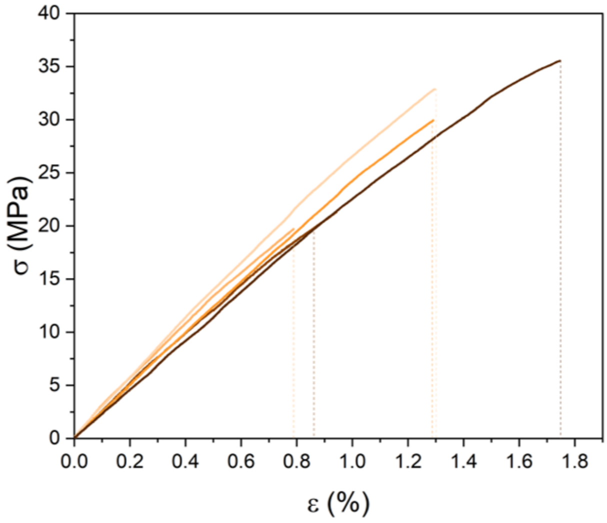

E (GPa)	σ_{max} (MPa)	ε_{break} (%)
2.53 ± 0.19	27.87 ± 7.04	1.21 ± 0.37

Figure S 3: Stress-strain curves of the five DGEBA-DETA specimens. The table shows the results in terms of elastic modulus (E), stress and elongation at break (σ_{max} and ε_{break}).

S4. CFRP characterization, with DGEBA-DETA polymeric matrix

The CF-reinforced composite (with DGEBA-PACM and DGEBA-DABP as polymeric matrix) was obtained through vacuum-bag impregnation, cured with the same thermal cycles. Four layers of 0°/0° unidirectional CFs, impregnated with the epoxy-amine resins, and cured with the same curing cycles reported for the thermoset specimens (Table S1).

However, DGEBA-DETA CFRPs was obtained through vacuum infusion and cured with the same thermal cycle reported for the resin. One layer of 0°/90° mat CFs (Figure S4) was impregnated with DGEBA-DETA resin, using conventional vacuum infusion equipment and obtaining the corresponding CFRPs. A composite with a fiber content around 50%wt (by thermo-gravimetric analysis (TGA) reported in Figure S5) was obtained; good fiber impregnation was confirmed by scanning electron microscopy (SEM) analysis (Figure S6).

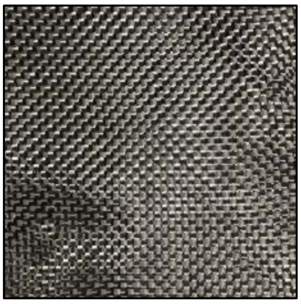

Figure S 4: One layer of virgin 0°/90° mat CFs.

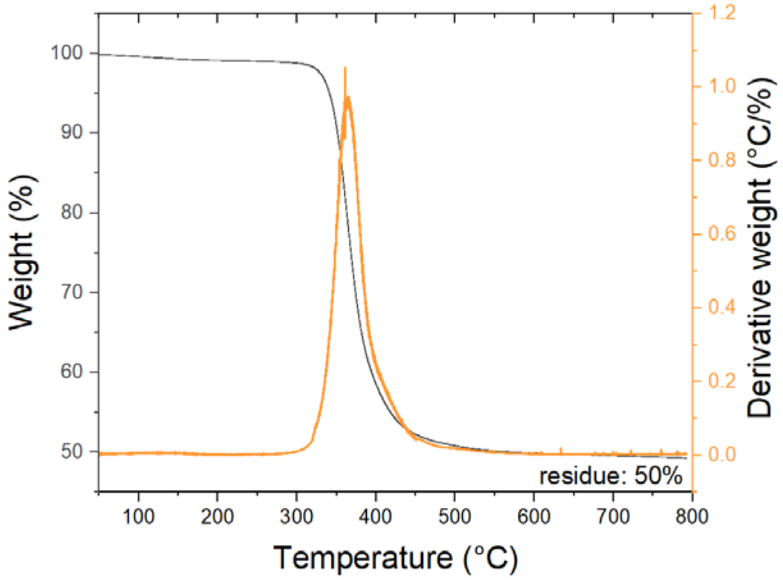

Figure S 5: TGA and DTGA curves of DGEBA-DETA CFRP, to evaluate the fiber content.

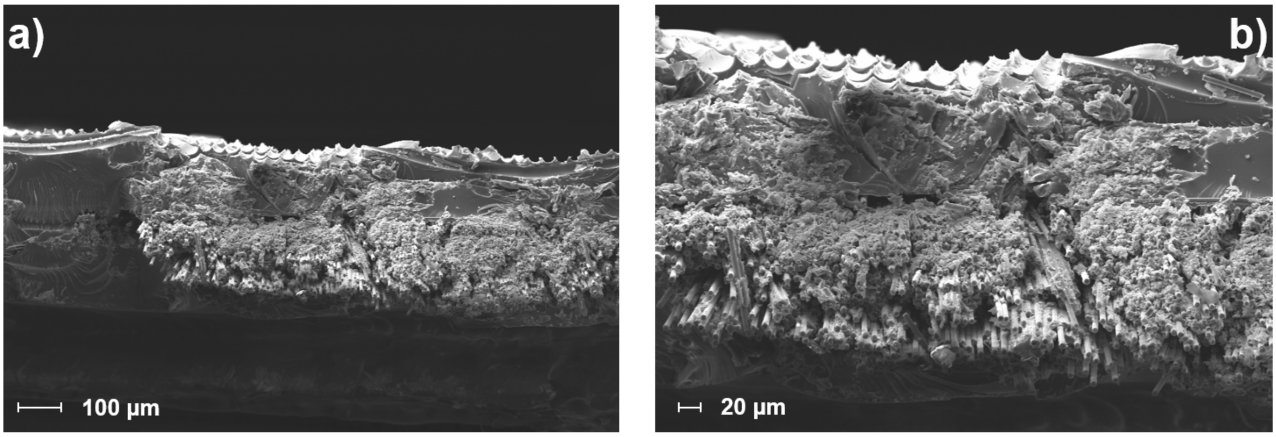

Figure S 6: SEM image of the one layer CF mat (0°/90°) with DGEBA-DETA polymeric matrix, to verify the correct fiber impregnation.

Tensile tests were performed on DGEBA-DETA CFRP using a INSTRON 5900R 4505 dynamometer equipped with a 100 kN load cell, applying a displacement rate of 2 mm min^{-1} at 25 °C, to determine the mechanical properties (ISO 527). The deformation was detected through a long-stroke extensometer. Five rectangular shaped samples with a gauge length of 80 mm, 15 mm width, and thickness of 0.2 mm were tested. The elastic modulus (E), determined from the slope of the curve between 0.05% and 0.25% of the total strain, strength and elongation at break (σ_{max} and ε_{break}) were evaluated and reported in Figure S7:

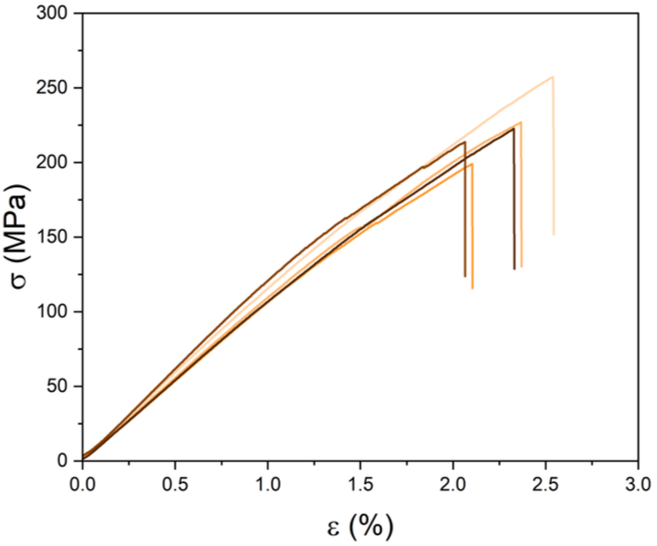

E (GPa)	σ_{max} (MPa)	ε_{break} (%)
11.1 ± 0.63	224.0 ± 21.47	2.28 ± 0.19

Figure S 7: Stress-strain curves of the CFRP specimens with one layer of mat CFs (0°/90°) and DGEBA-DETA network. The table shows the results in terms of elastic modulus (E), stress and elongation at break (σ_{max} and ε_{break}).

S5. Solvent and Lewis acid selection

Different selection criteria were employed to determine the suitable solvent for the solvolysis reaction, namely safety, boiling temperature (Tb) and biodegradability (defined according to OECD criteria) (S1). Table S2 summarizes the characteristics of the carboxylic acids under investigation:

Table S 2: Solvents characteristics in terms of boiling temperature (Tb) and biodegradability.

Solvent	Tb (°C)	Biodegradable
Valeric acid	185	readily
Caproic acid	205	readily
Heptanoic acid	223	readily
Pelargonic acid	254	readily
2-Ethylhexanoic acid	228	readily
Oleic acid	360	readily
Lauric acid	299	readily

Moreover, the thermodynamic affinity and swelling capabilities of the investigated solvents with the aliphatic epoxy-amine cured thermoset is evaluated by means of Hansen solubility theory (S2). The solubility parameters of the carboxylic acids were computed exploiting the group contributions method of Van Krevelen and Hoftyzer (3). The results in terms of Hansen solubility parameters (δd, δp and δh) are reported in Table S3:

Table S 3: Computed Hansen solubility parameters of the carboxylic acids under investigation.

Solvent	δd ($\sqrt{\mathrm{J/cm^2}}$)	δp ($\sqrt{\mathrm{J/cm^2}}$)	δh ($\sqrt{\mathrm{J/cm^2}}$)
Valeric acid	15.0	4.1	10.3
Caproic acid	15.0	4.1	9.4
Heptanoic acid	15.1	3.3	8.2
Pelargonic acid	16.0	3.0	7.8
2-Ethylhexanoic acid	15.8	2.6	7.9
Oleic acid	14.3	3.1	14.3
Lauric acid	16.2	4.0	7.4

Considering the DGEBA-DETA epoxy resin, the related Hansen solubility parameters and the corresponding radius R are reported in Table S4 (4,5):

Table S 4: Hansen solubility parameters of the DGEBA-DETA epoxy resin.

	δd ($\sqrt{\mathrm{J/cm^2}}$)	δp ($\sqrt{\mathrm{J/cm^2}}$)	δh ($\sqrt{\mathrm{J/cm^2}}$)	R
DGEBA-DETA	19.5	19.4	4.3	19.9

Consequently, the interaction sphere of the epoxy resin can be drawn in the Hansen space (Figure S8), together with the different solvents. All the good solvents would be inside the interaction sphere. Moreover, the solvents with the lowest relative energy difference (RED = R_a/R) can be considered as the most thermodynamically affine.

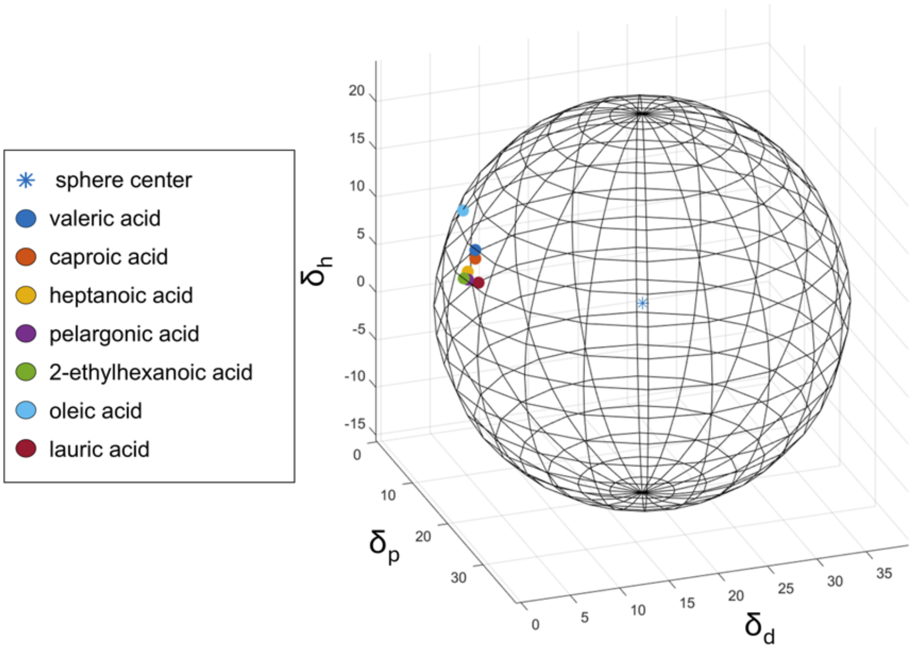

Solvent	RED
valeric acid	0.9435
caproic acid	0.9301
heptanoic acid	0.9447
pelargonic acid	0.9148
2-ethylhexanoic acid	0.9389
oleic acid	1.0956
lauric acid	0.8579

Figure S 8: Hansen solubility sphere of the DGEBA-DETA thermoset, with the relative position of the investigated solvents and the computed RED values.

S6. Organometallic coordination complexes characterizations

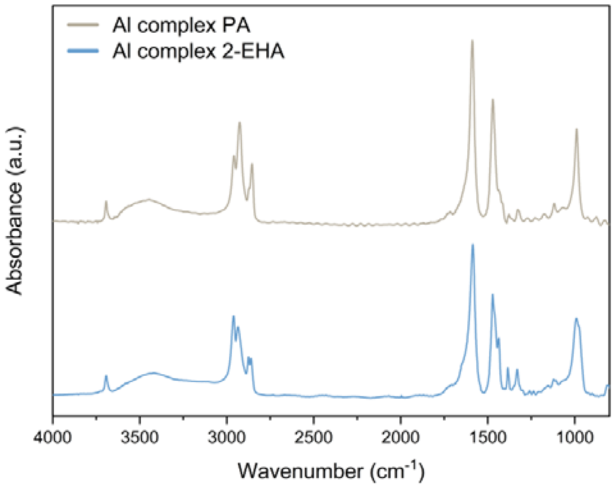

Figure S 9: FTIR spectra of organometallic coordination complex, recovered after precipitation and filtration at the end of the solvolysis process with PA and 2-EHA as carboxylic acid.

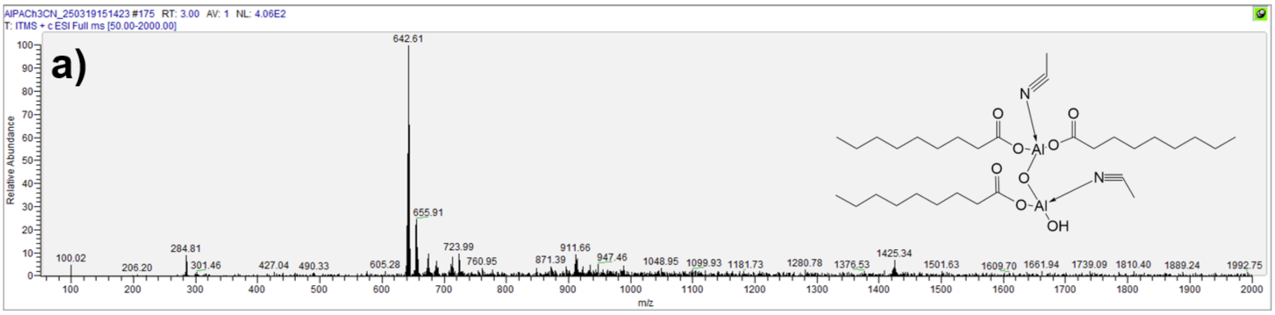

b)

C %	H %	N %
59.70	9.88	0

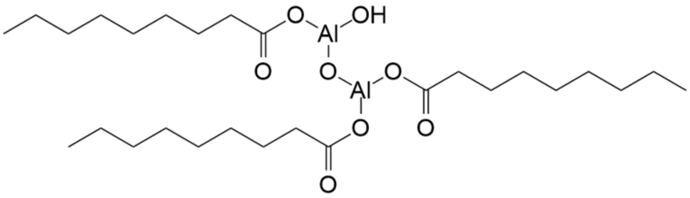

Figure S 10: a) ESI-MS of the Al complex PA in acetonitrile, the coordination structure between the compound the solvent is reported; b) (left) elemental analysis CHN % of the Al complex PA, (right) hypothesized chemical structure of the Al complex.

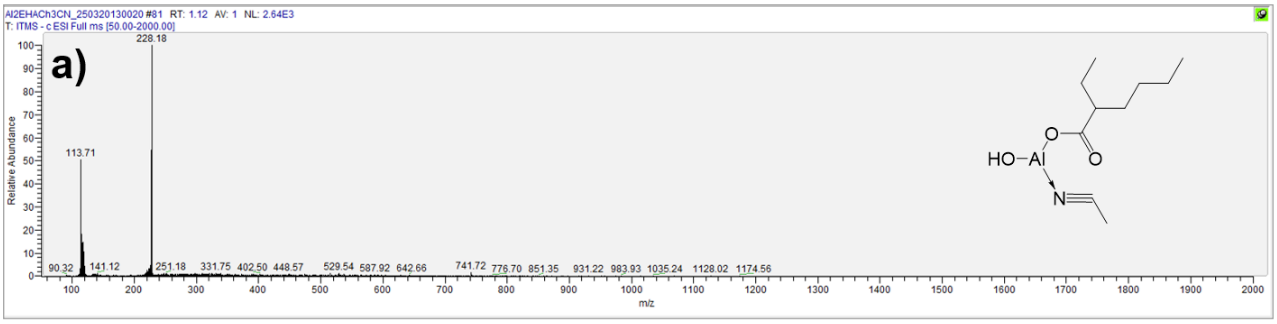

b)

C %	H %	N %
57.53	9.50	0

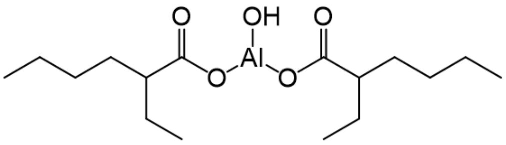

Figure S 11*:* a) ESI-MS of the Al complex 2-EHA in acetonitrile, the coordination structure between the compound the solvent is reported; b) (left) elemental analysis CHN % of the Al complex 2-EHA, (right) hypothesized chemical structure of the Al complex.

The organometallic complex, precipitated as solid at the end of the solvolysis process and separated from the organic mixture by filtration, was mixed with neat $AlCl_3$ in different weight percentages (as reported in Table S5) in order to determine the optimal ratio between the two components.

Table S 5: Catalytic effects of different weight percentage proportions of $AlCl_3$ and organometallic coordination complex on the D_r and on the pH of the resulting organic solution. The solvolysis was performed at 200 °C for 6 h on 1 g of DGEBA-DETA epoxy resin, with 10 g of 2-EHA or PA (depending on the composition of the organometallic coordination complex) and 1.1 g of catalytic mixture (relative composition reported in the table).

	**Organometallic coordination complex (%)**	**$AlCl_3$ (%)**	**D_r (%)**	**pH**
1	0	100	> 99	< 1
2	50	50	> 99	3 - 4
3	80	20	> 99	3 - 4
4	90	10	> 99	4 - 5
5	100	0	swelling	5

S7. Mild solvolysis process of thermoset and CFRP

Table S 6: Influence of epoxy resin concentration, catalyst concentration, reaction time and reaction temperature on D_r. The concentration of the DGEBA-DETA epoxy resin was calculated with respect to the total quantity of solvent (2-EHA or PA) in the reaction, while the concentration of the catalyst with respect to the total mass of reactants (i.e., solvent and thermoset). The reaction conditions were tested on both solvents (2-EHA or PA) with no differences.

	**Epoxy resin concentration (*%wt*)**	**Modified-catalyst concentration (*%wt*)**	**Reaction time (h)**	**Reaction temperature (°C)**	**D_r (%)**
1	5	10	6	200	> 99
2	10	10	6	200	> 99
3	10	5	8	200	60
4	10	5	12	200	> 99
5	10	5	12	170	44
6	20	5	12	200	75

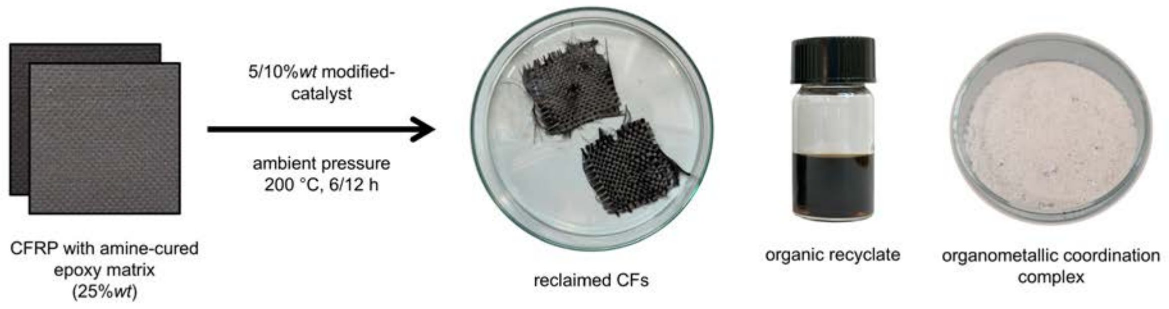

Figure S 12: Chemical recycling of CFRP, constituted of DGEBA-DETA polymeric matrix.

S8. Characterization of the organic fraction

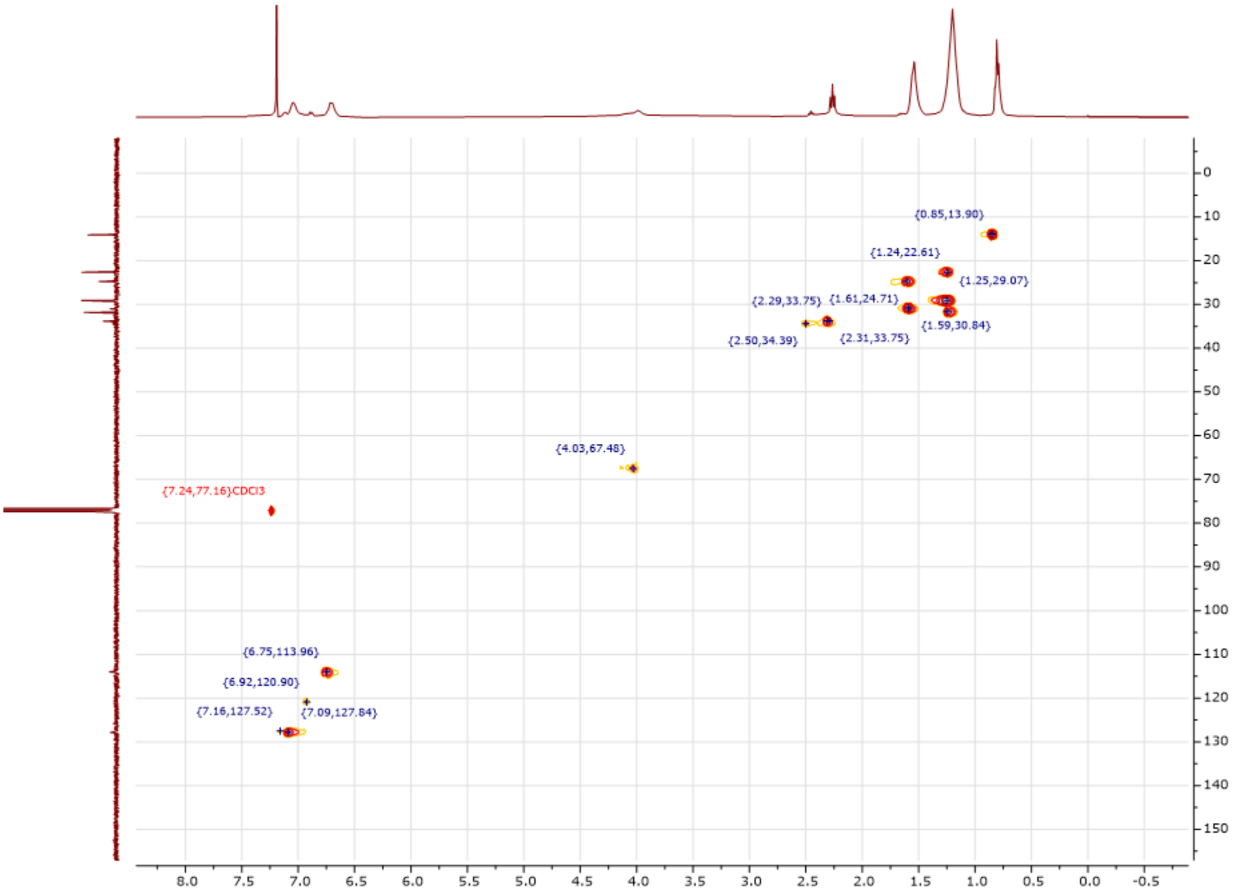

Figure S 13*:* HSQC ^{1}H – ^{13}C NMR spectra of the oligomeric fraction at 25 °C in $CDCl_3$.

^{1}H NMR (400 MHz, $CDCl_3$) δ 7.14 – 7.09 (m, 4H), 6.94 – 6.76 (m, 4H), 412 – 3.96 (m, 6H), 2.51 (t, J=7.5 Hz, 1H), 2.31 (t, J=7.5 Hz, 5H), 1.59 (s, 13H), 1.27 (d, J=12.9 Hz, 32H), 0.86 (t, J=6.7 Hz, 10H).

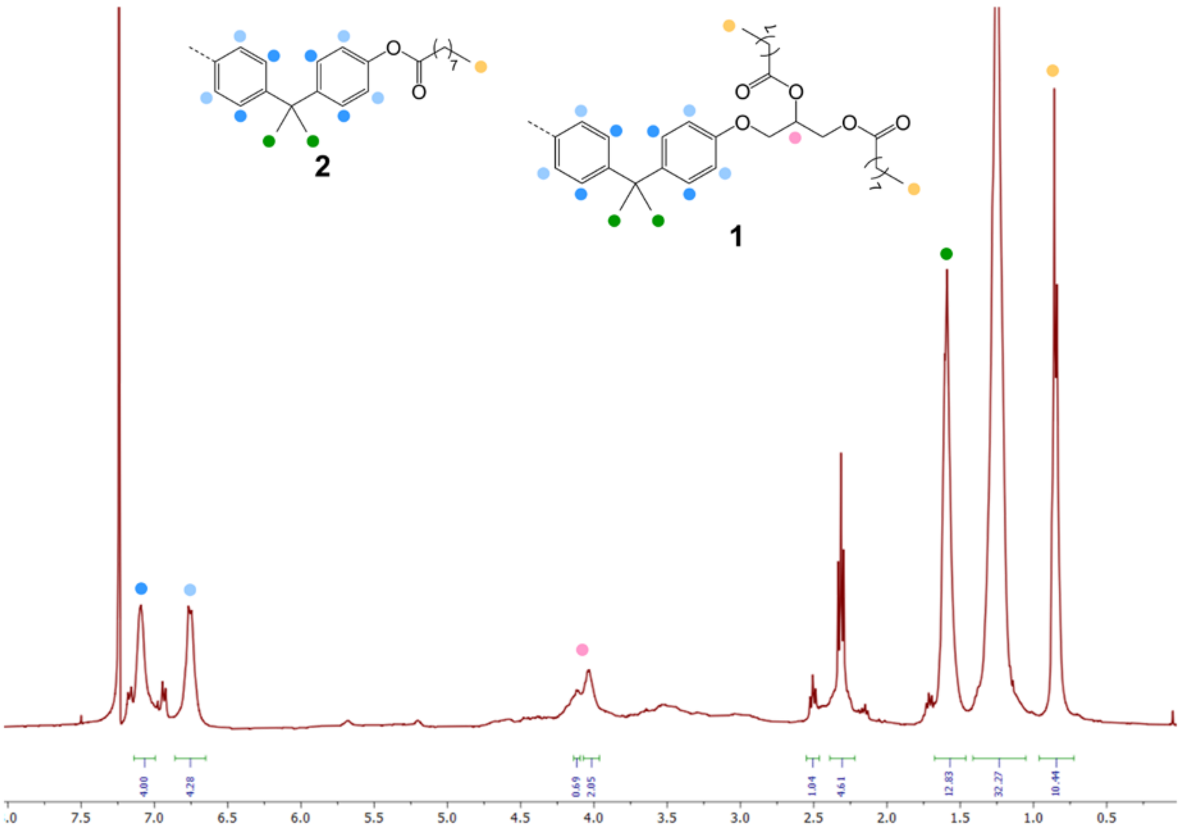

Figure S 14: ^{1}H NMR spectrum of the oligomeric fraction at 25 °C in $CDCl_3$, with tentative attribution of the peaks characteristic of the two main oligomeric structures.

^{13}**C NMR** (101 MHz, $CDCl_3$) δ 178.06, 127.83, 113.99, 33.82, 31.79, 30.98, 29.20, 29.09, 24.74, 22.62, 14.07.

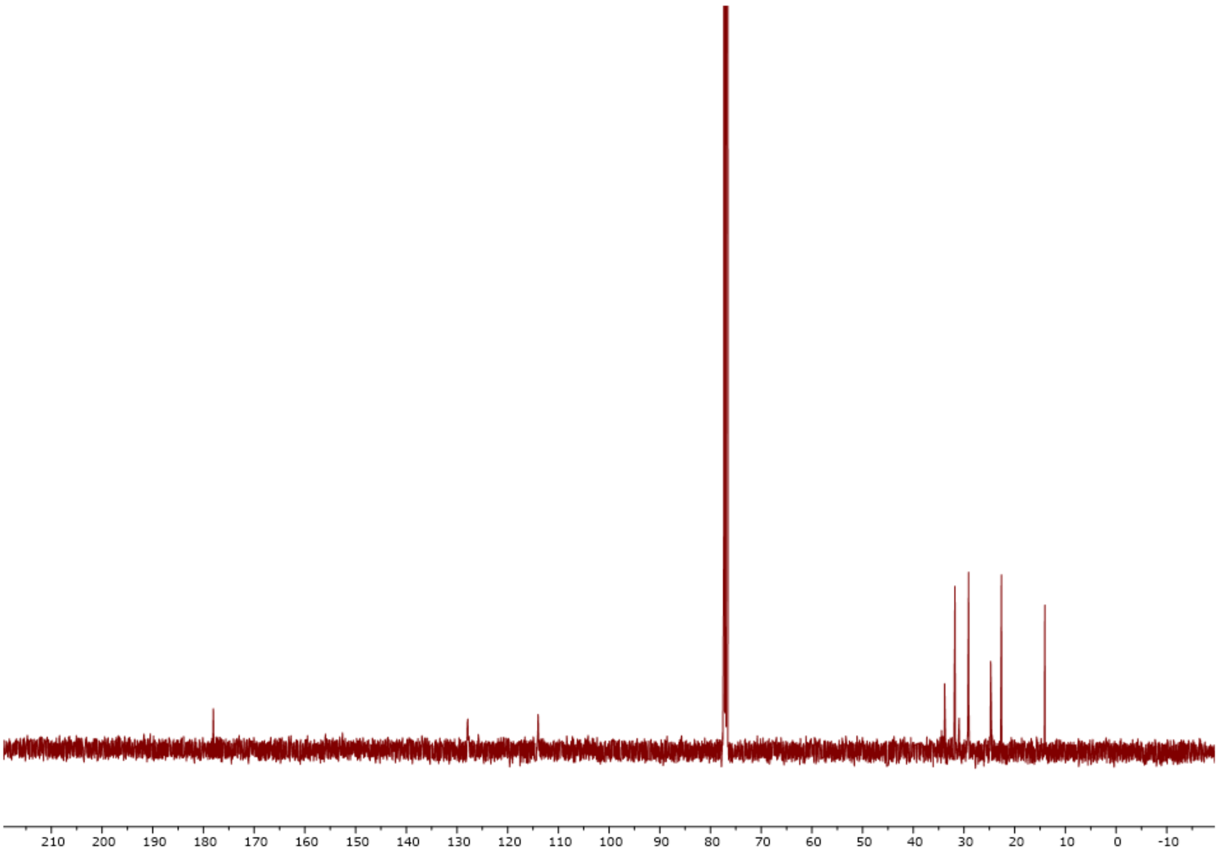

Figure S 15: ^{13}C NMR spectrum of the oligomeric fraction at 25 °C in $CDCl_3$.

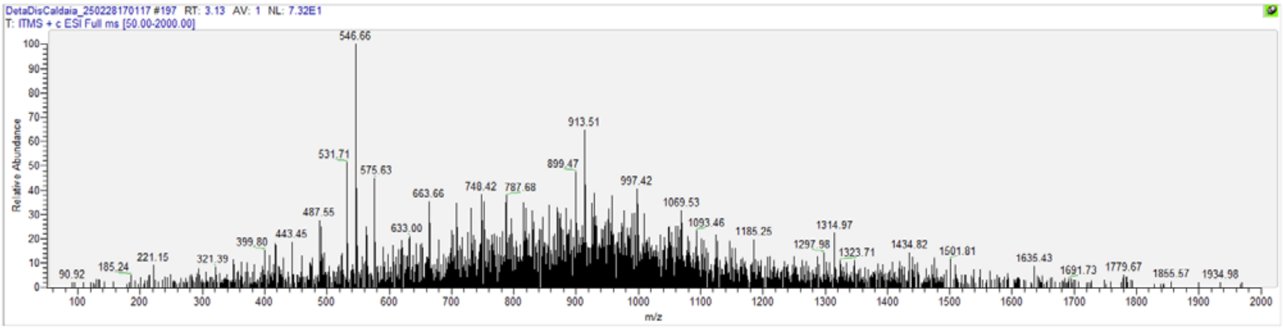

Figure S 16: ESI-MS in acetonitrile of the organic fraction recovered at the end of the work-up procedures.

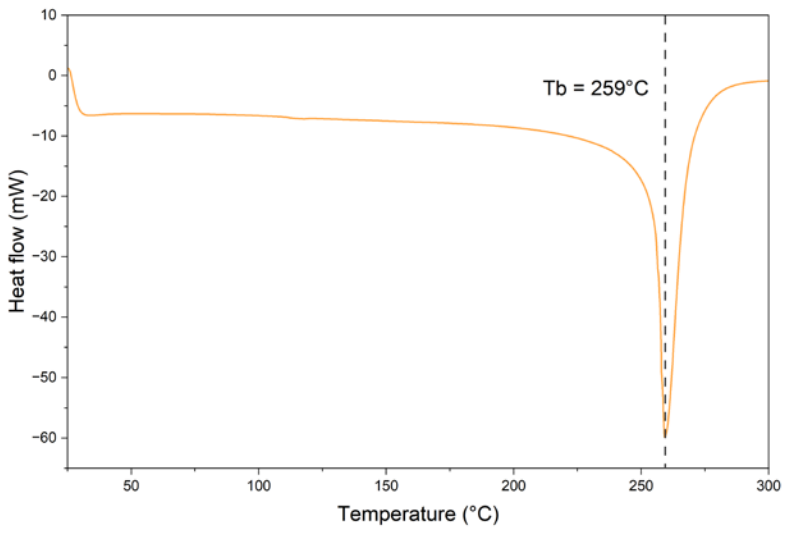

Figure S 17: DSC curve of the oligomeric fraction recovered at the end of the solvolysis process. The boiling temperature (Tb) was identified as endothermic peak in the first heating run of the thermograph.

The viscosity of the oligomeric fraction was evaluated at 20 °C by rotational rheology with a 20-mm-diameter plate and a Peltier plate temperature controller (TA Instruments DHR-2 rheometer). The organic recyclate exhibited a distinctive Newtonian plateau at high shear rates, with a shear viscosity of 16 mPa·s.

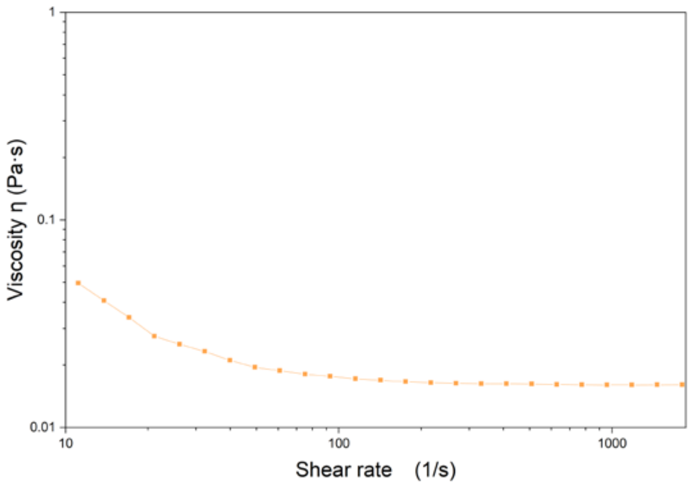

Figure S 18: Viscosity curve of the oligomeric fraction recovered at the end of the solvolysis process.

S9. Characterization of the recovered CFs

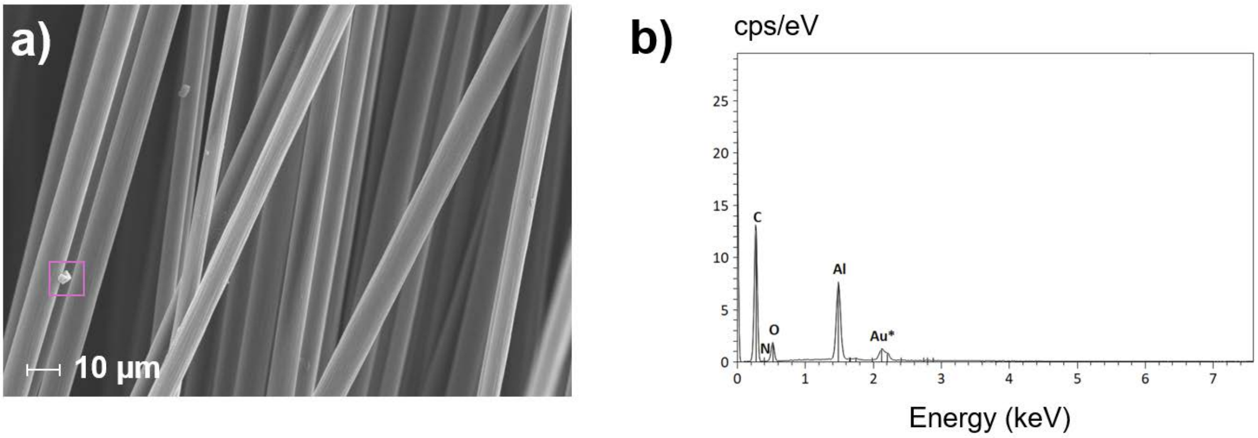

Figure S 19: a) SEM image of rCFs at 2.00x magnification; b) EDX analysis of the residue on the fibers surface, specifically the residue highlighted in a). The Au* peak is related to the gold plating process before the analysis.

The Weibull distribution was employed to evaluate the reliability of the model in terms of time to failure. The Weibull modulus *m* was determined graphically as the slope of the ln[ln(1/(1-Pf))] vs. ln(σ_{max}) plot, where σ_{max} is the maximum tensile strength and Pf is probability of failure when the samples are ranked from weakest to strongest, estimated according to:

$$Pf = \frac{i}{N+1}$$

where i is the rank of the ith specimen and N is the total number of samples (S6).

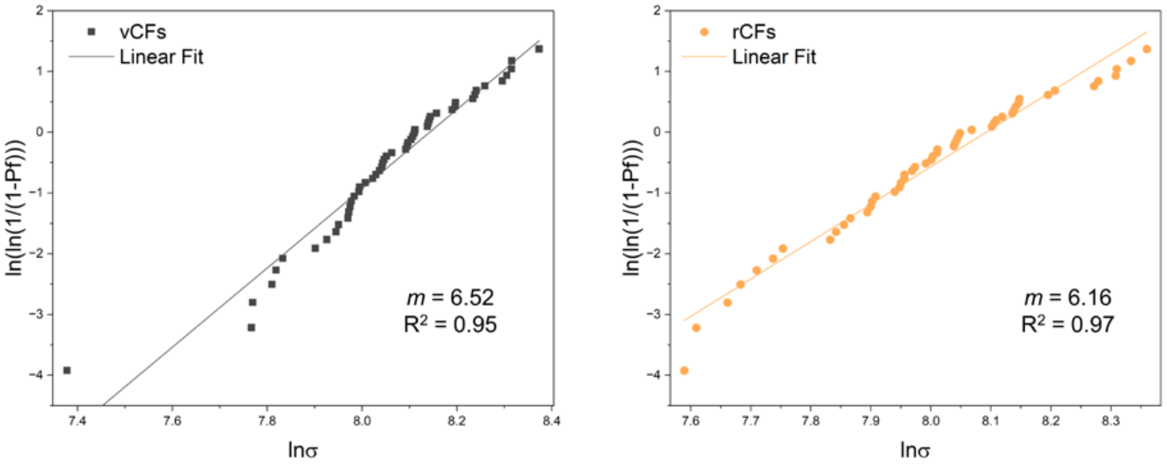

Figure S 20: Weibull plot of virgin (left) and recycled(right) CFs. The Weibull modulus (*m*) was obtained as slope of the linear fit.

The Weibull analysis showed that the Weibull modulus *m* of vCFs is comparable to that of rCFs, indicating a similar statistical distribution of tensile strength for both materials. This result further suggests that the solvolysis treatment did not introduce significant additional variability or defects, leading to recycled fibers with high strength, good uniformity, and high purity.

S10. Process validation: solvolysis process on different model epoxy resins

The reported chemical recycling process was tested on two different epoxy-amine systems: DGEBA-PACM (Tg = 180 °C) and DGEBA-DABP (Tg = 157 °C). Clearly, slight modifications on the process parameters were necessary to obtain complete degradation of the resins, due to the different structures and chemical properties with respect to the DGEBA-DETA system. The best degradation conditions are reported in Table S7 (line 1 and line 6):

Table S 7: Process parameters tested on DGEBA-PACM and DGEBA-DABP thermosets, to individuate the best operating conditions. . The concentration of the resin was calculated with respect to the total quantity of solvent (2-EHA or PA) in the reaction, while the concentration of the catalyst with respect to the total mass of reactants (i.e., solvent and thermoset). The reaction conditions were tested on both solvents (2-EHA or PA) with no differences.

	**Epoxy resin concentration (*%wt*)**	**Modified-catalyst concentration (*%wt*)**	**Reaction time (h)**	**Reaction temperature (°C)**	**D_r (%)**
1	5	10	18	200	> 99
2	10	7	18	200	75
3	10	10	24	190	62
4	10	10	18	200	92
5	10	7	12	210	93
6	10	7	24	210	> 99

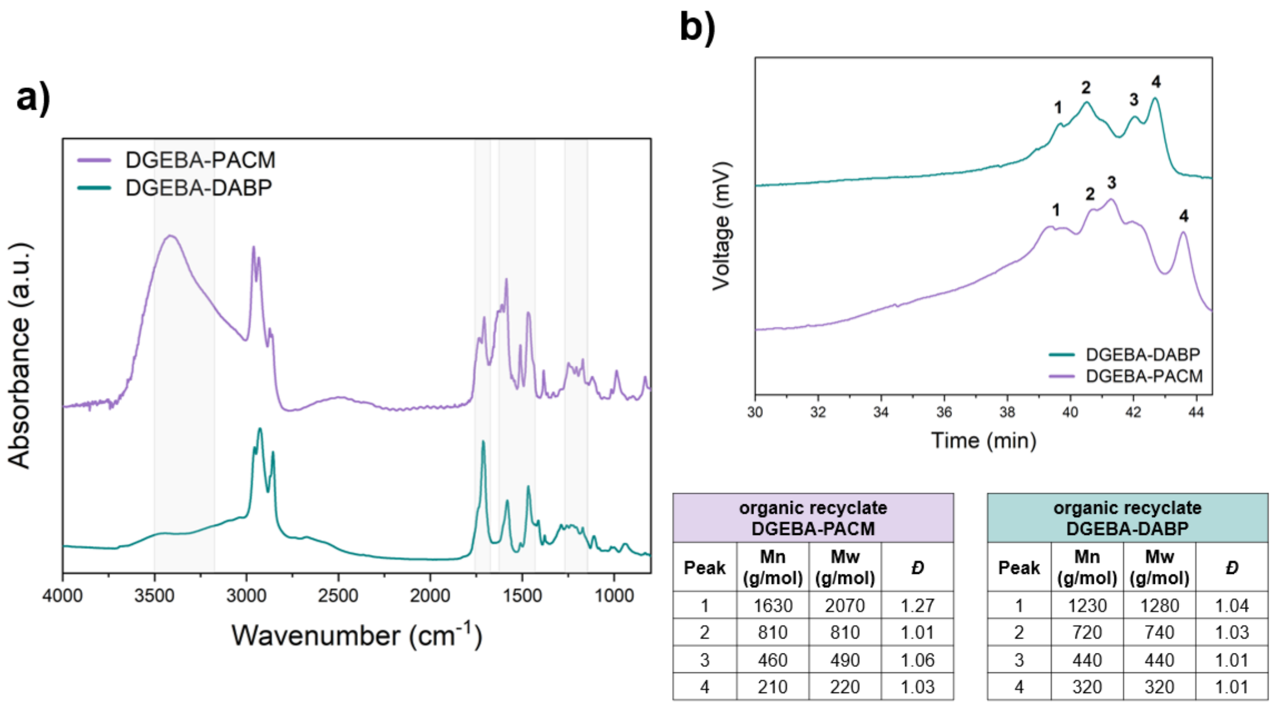

organic recyclate DGEBA-PACM			
Peak	Mn (g/mol)	Mw (g/mol)	Đ
1	1630	2070	1.27
2	810	810	1.01
3	460	490	1.06
4	210	220	1.03

organic recyclate DGEBA-DABP			
Peak	Mn (g/mol)	Mw (g/mol)	Đ
1	1230	1280	1.04
2	720	740	1.03
3	440	440	1.01
4	320	320	1.01

Figure S 21: a) FTIR spectra of the recovered organic recyclates recovered at the end of the solvolysis process of DGEBA-PACM and DGEBA-DABP; b) GPC analysis of the organic recyclate.

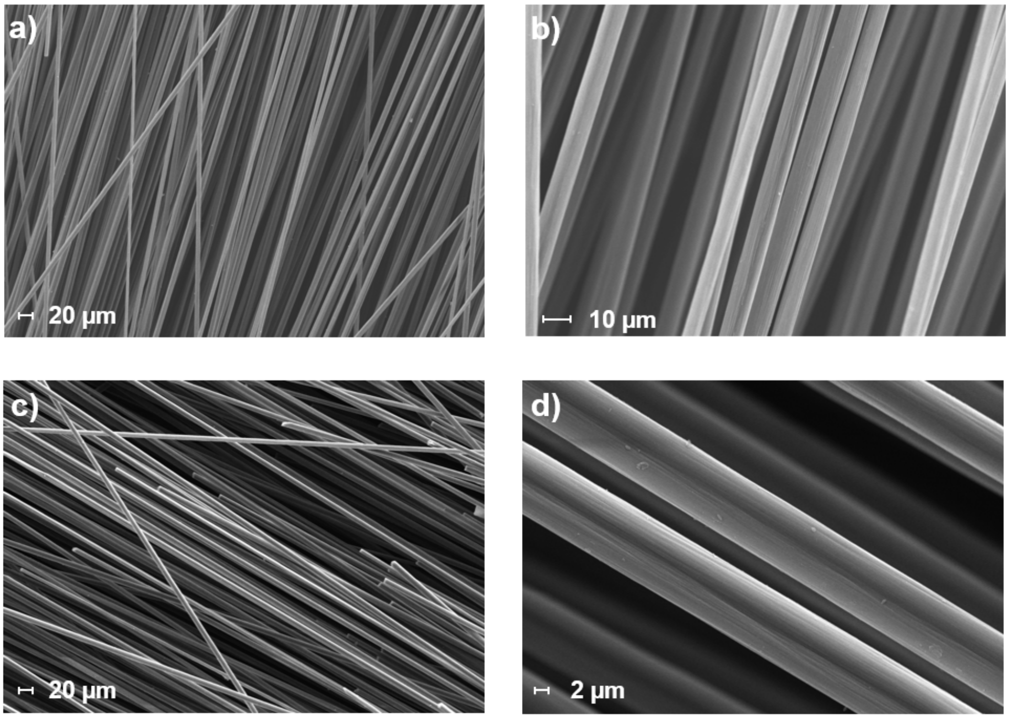

Figure S 22:a), b) SEM images of rCFs from DGEBA-PACM polymeric matrix, at 500 X and 2.00 kX magnifications; c), d) SEM images of rCFs from DGEBA-DABP polymeric network, at 500 X and 5.00 kX magnifications.

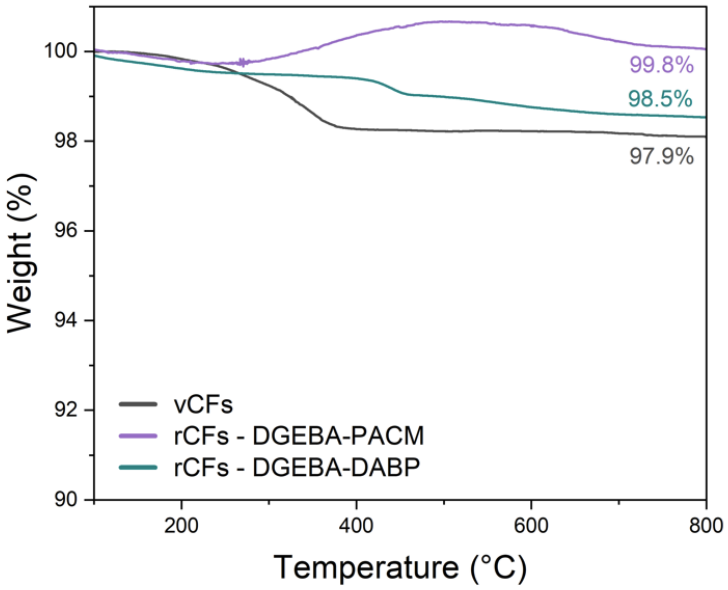

Figure S 23: TGA analysis in nitrogen atmosphere of virgin and recovered CFs of DGEBA-PACM and DGEBA-DABP polymeric networks.

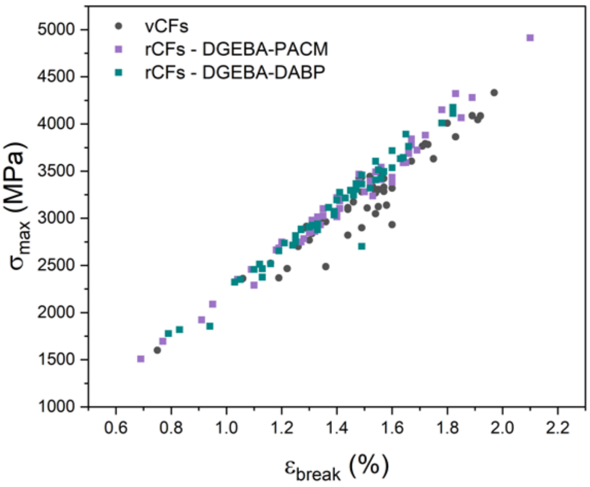

	E (GPa)	σ_{max} (MPa)	ε_{break} (%)	d (µm)
vCFs	211.34 ± 10.67	3200 ± 520	1.50 ± 0.23	7.55 ± 0.38
rCFs – DGEBA-PACM	220.10 ± 6.07	3170 ± 660	1.42 ± 0.28	7.59 ± 0.38
rCFs – DGEBA-DABP	221.11 ± 8.12	3070 ± 565	1.38 ± 0.24	7.33 ± 0.40

Figure S 24: Distribution plot of maximum stress and maximum strain of virgin and recycled CFs. The table reports elastic modulus (E), stress and elongation at break (σ_{max} and ε_{break}, respectively) and fiber diameter (d) of virgin and recycled CFs, obtained through single-fiber tensile tester.

S11. Second-generation CFRP

Different amount of the reclaimed oligomeric fraction were added in the initial formulation of the DGEBA-DETA resin. The effect of the content was evaluated through Tg (DSC curve reported in Figure S25) and gel content. The gel content results > 99% in all the three case studies.

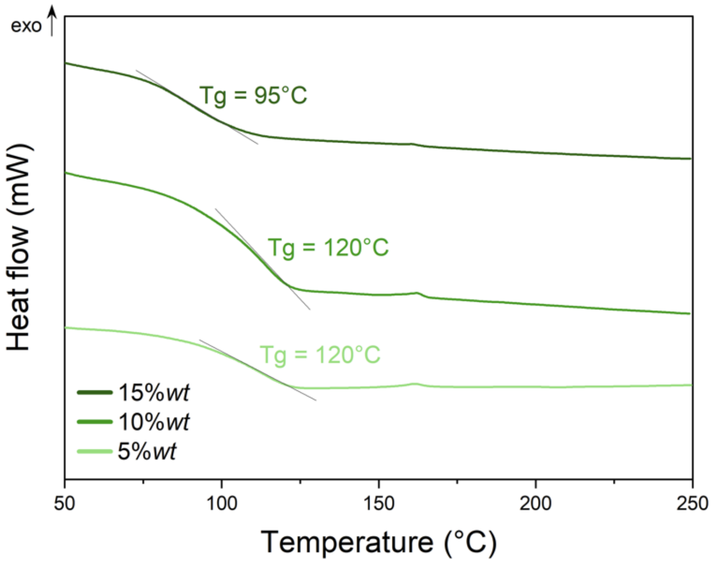

Figure S 25: DSC curves of the epoxy-amine resin with 5,10 and 15%*wt* of oligomeric fraction, for glass transition temperature (Tg) identification as inflection point in the second heating run of the thermographs.

Tensile tests were performed on DGEBA-DETA resin with 10%*wt* of the oligomeric fraction using a Zwick/Roell BT-FR010TH.A50 tensile testing machine equipped with a 10 kN load cell, applying a displacement rate of 1 mm min-1 at 25 °C. The deformation was detected through a long-stroke extensometer. The elastic modulus (E), determined from the slope of the curve between 0.05% and 0.15% of the total strain, strength and elongation at break (σ_{max} and ε_{break}) were evaluated (Figure S26):

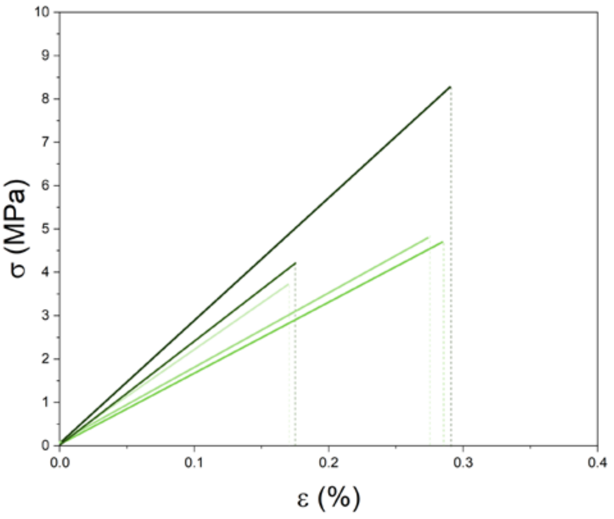

E (GPa)	σ_{max} (MPa)	ε_{break} (%)
2.12 ± 0.47	5.12 ± 1.74	0.24 ± 0.06

Figure S 26: Stress-strain curves of the second-generation resin with 10%*wt* oligomers. The table shows the results in terms of elastic modulus (E), stress and elongation at break (σ_{max} and ε_{break}).

The second-generation CFRP was fabricated though vacuum-bag impregnation, with one layer mat of vCFs or rCFs impregnated with DGEBA-DETA resin with 10%*wt* of the oligomeric fraction.

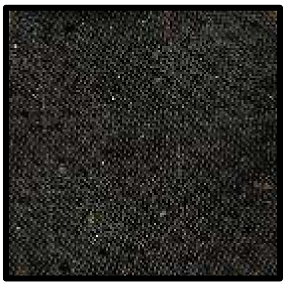

Figure S 27: Second-generation CFRP (4 x 4 x 0.2 cm), fabricated through vacuum-bag impregnation with one layer rCFs mat and DGEBA-DETA resin with 10%*wt* oligomeric fraction, recovered after the solvolysis process.

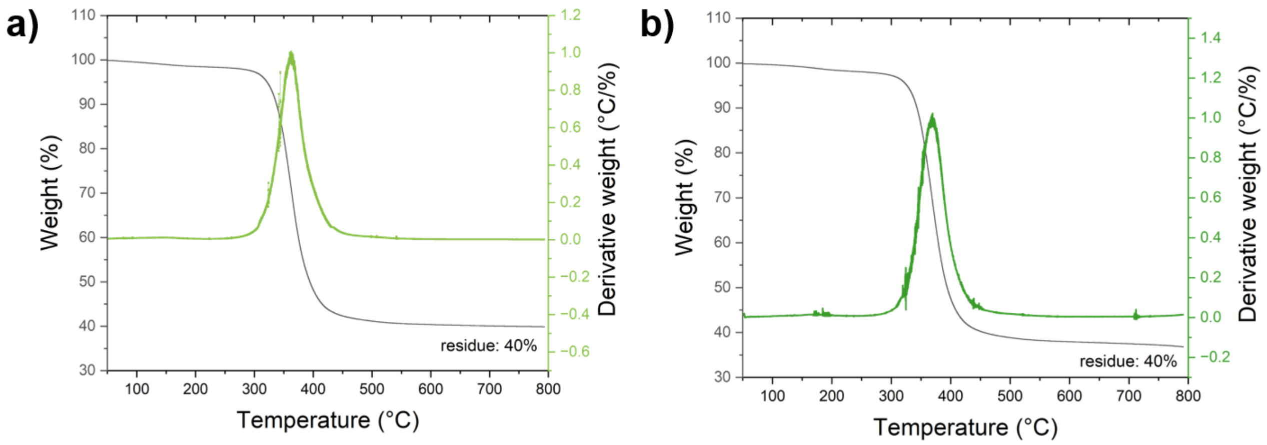

Figure S 28: TGA and DTGA curves of the second-generation CFRP to evaluate the fiber content: a) CFRP with vCFs and 10%*wt* oligomeric fraction; b) CFRP with rCFs and 10%*wt* oligomeric fraction.

Tensile tests were performed on second-generation CFRP using a INSTRON 5900R 4505 dynamometer equipped with a 100 kN load cell, applying a displacement rate of 0.1 mm min^{-1} at 25 °C, to determine the mechanical properties according to ISO 527, namely the elastic modulus (E), determined from the slope of the curve between 0.05% and 0.25% of the total strain, strength and elongation at break (σ_{max} and ε_{break}).

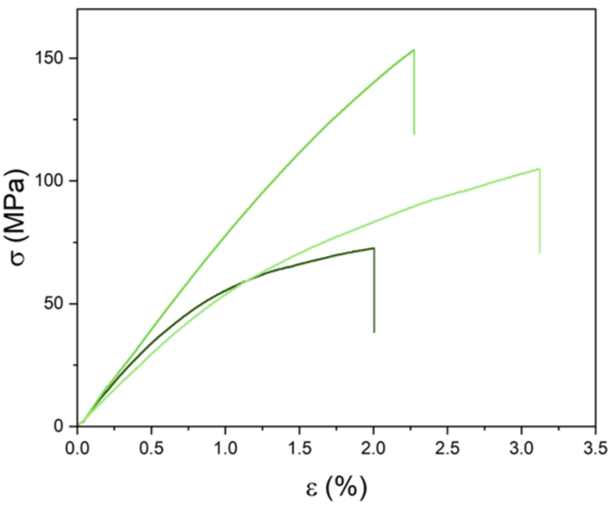

E (GPa)	σ_{max} (MPa)	ε_{break} (%)
7.4 ± 1.09	110.2 ± 40.67	2.46 ± 0.58

Figure S 29: Stress-strain curves of second-generation CFRP specimens with one layer of mat rCFs (0°/90°) and DGEBA-DETA network incorporating 10%wt of organic recyclates. The table show the results of tensile testing in terms of elastic modulus (E), maximum stress (σ_{max}) and elongation at break (ε_{break}).

S12. End-of-life components characterization

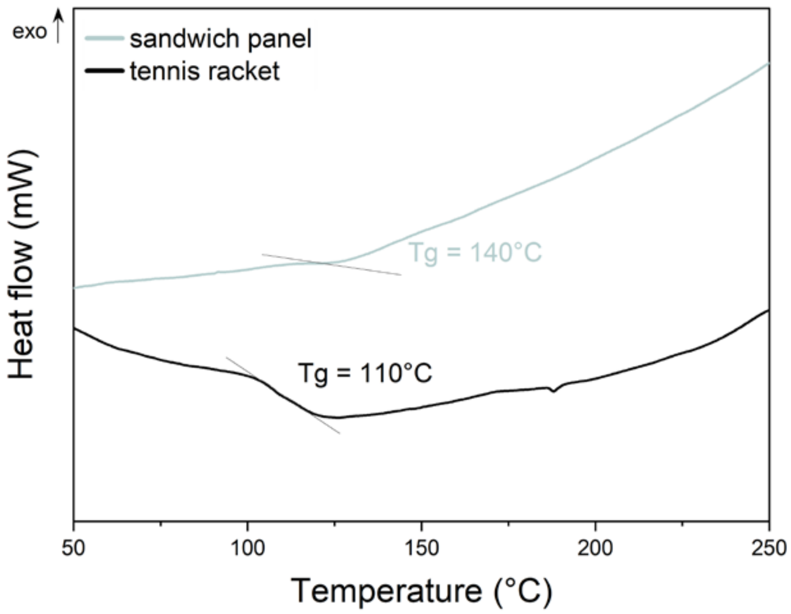

Figure S 30: DSC curves of the end-of-life fragments for glass transition temperature (Tg) identification as inflection point in the second heating run of the thermographs.

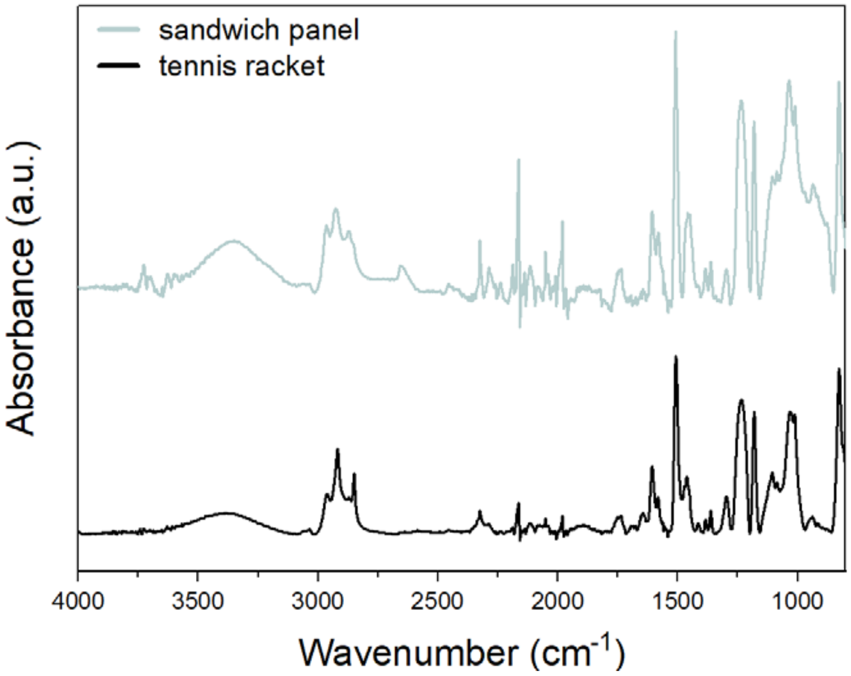

Figure S 31: FTIR of tennis racket and sandwich panel fragments.

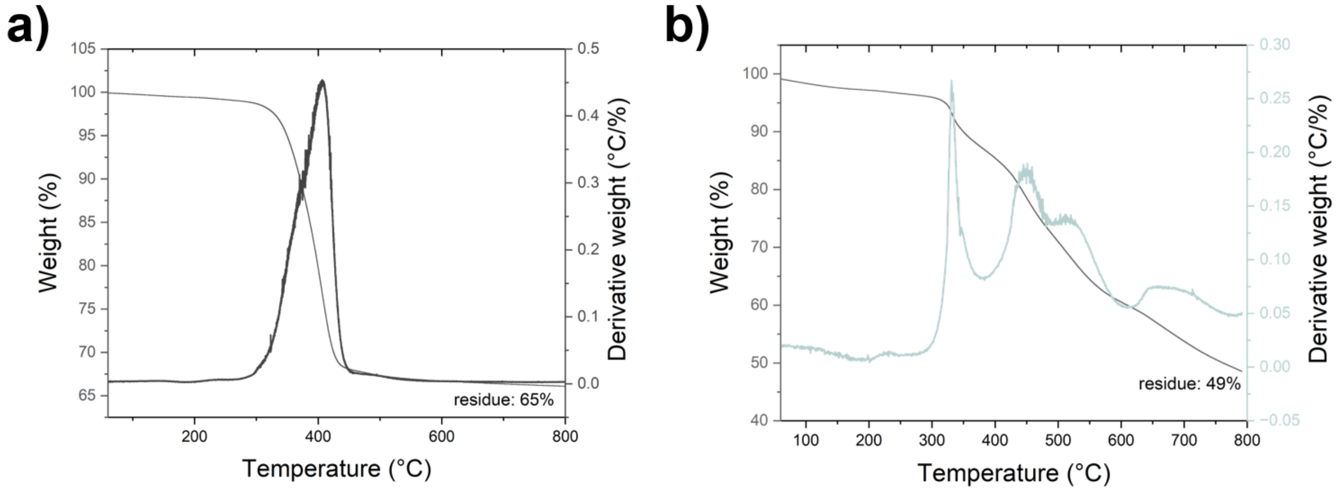

Figure S 32: TGA and DTGA curves of the end-of-life fragments: a) tennis racket, b) sandwich panel.

S13. Calculation of material circularity indicators (MCI)

Based on the methodology originally proposed by the Ellen MacArthur foundation in 2015 and further updated in 2019 (https://www.ellenmacarthurfoundation.org/material-circularity-indicator), the material circularity indicator (MCI) is built from a combination of three product characteristics: mass of employed raw virgin material (V), mass of non-recoverable waste (W) and the utility factor (X), which considers the duration and the use of the product under investigation. The MCI indicates how circular a specific material or product is, going from fully linear (MCI = 0.1) to fully circular (MCI = 1). It can be calculated using the following expression:

$$MCI = 1 - LFI\left(\frac{0.9}{X}\right)$$

where LFI represents the linear flow index.

Table S8 lists the main terms and variables (with corresponding definitions) used for the evaluation of the MCI, as reported in the Ellen MacArthur Foundation methodology.

Table S 8: List of symbols and definitions for the calculation of MCI, according to the Ellen MacArthur Foundation methodology.

Symbol	Definition
M	Mass of a product
F_R	Fraction of mass of a product's feedstock from recycled sources
F_U	Fraction of mass of a product's feedstock from reused sources
F_S	Fraction of a product's biological feedstock from Sustained Production. Biological material that is recycled or reused is captured as recycled or reused material, not biological feedstock.
V	Material that is not from reuse, recycling or, for the purposes of this methodology, biological materials from Sustained Production.
C_R	Fraction of mass of a product being collected to go into a recycling process
C_U	Fraction of mass of a product going into component reuse
C_C	Fraction of mass of a product being collected to go into a composting process
C_E	Fraction of mass of a product being collected for energy recovery where the material satisfies the requirements for inclusion.
W_0	Mass of unrecoverable waste through a product's material going into landfill, waste to energy and any other type of process where the materials are no longer recoverable
E_C	Efficiency of the recycling process used for the portion of a product collected for recycling
W_C	Mass of unrecoverable waste generated in the process of recycling parts of a product
E_F	Efficiency of the recycling process used to produce recycled feedstock for a product
W_F	Mass of unrecoverable waste generated when producing recycled feedstock for a product
W	Mass of unrecoverable waste associated with a product
L	Actual average lifetime of a product
L_{av}	Average lifetime of an industry-average product of the same type
U	Actual average number of functional units achieved during the use phase of a product
U_{av}	Average number of functional units achieved during the use phase of an industry average product of the same type

The circularity potential of the chemical recycling process proposed in this work was estimated by calculating the MCI for two key elements of the process, namely the reaction solvent and the catalyst. Indeed, in the solvolysis process developed here, it was shown that they can both be recovered (at a rate of at least 90%, considering possible losses during the work-up procedures) and reused for multiple solvolysis cycles without affecting the yield of the chemical recycling reaction, ultimately reducing the need of virgin feedstock materials (*viz*., solvent and catalyst).

Furthermore, an additional assessment of the potential impact of the solvolysis process on the circularity of CFRPs was also carried out, by calculating the MCI for second-generation CFRP materials incorporating rCFs reclaimed from the solvolysis process as only reinforcement, and a fraction (10%*wt*) of the organic recyclate recovered from the chemical recycling process in the resin formulation.

S13.1 MCI of the reaction solvent

Based on the experimental results reported in this work (see main text), the following assumptions can be made. Considering 10 g of CFRP, the optimized solvent quantity for the solvolysis reaction is 33.3 g (*i.e.*, 30%*wt* of sample with respect to the solvent): consequently, M = 33.3 g. As demonstrated in this work, the solvent used as feedstock material for a given chemical recycling process can be obtained as recycled product from a previous solvolysis step through vacuum distillation (considering a solvent recovery rate of at least 90%, including a conservative 10% of losses occurring during work-up procedures, E_F = 0.9). Indeed, it does not differ from its virgin counterpart (Figure S33). Assuming a replacement rate of 50%*wt* of the total amount of required virgin solvent, F_R = 0.5. Accordingly, the quantity of virgin material (V) necessary for the reaction under investigation is $V = M * (1 – F_R – F_U – F_S)$ = 16.7 g (F_U and F_S are both nil, as this calculation does not consider the presence of reused feedstock or feedstock from biological sources, for the sake of simplicity).

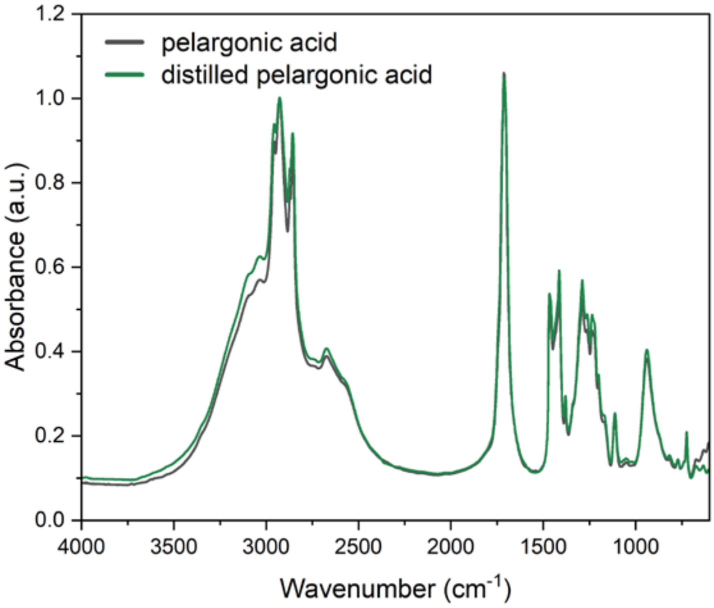

Figure S 33: FTIR of pelargonic acid vs distilled pelargonic acid recovered at the end of the solvolysis process.

At the end of the solvolysis process, the solvent can be further recycled through vacuum distillation (C_R = 1, as the whole of it will be recyclable, and so $W_0 = M * (1 – C_R – C_U – C_C – C_E) = 0$, since C_U, C_C and C_E are nil) for use in a subsequent process, still with 90% recycling efficiency (E_C = 0.9). Hence, the total amount of waste

produced during the solvolysis process (W) can be computed: $W = W_0 + (W_C + W_F)/2$, where $W_C = M * (1 - E_C) * C_R$ and $W_F = M * (1 - E_F) * F_R/E_F$. As a result, the LFI becomes:

$$LFI = \frac{V + W}{2M + \frac{W_F - W_C}{2}} = 0.29$$

Assuming $L/L_{av} = 1$ and $U/U_{av} = 1$, the utility factor (X, $X = (L/L_{av}) * (U/U_{av})$) is 1.

Based on these assumptions, the calculation of the MCI for the solvent (recycled and recyclable) used in the solvolysis process presented in this work yields $MCI_{solvent}$ = 0.74. Figure S34 shows a comparison in $MCI_{solvent}$ values between the fully-linear base-case scenario and the scenario characteristic of the process presented in this work, with an evident increase in the level of circularity (*viz*., $MCI_{solvent}$) for the solvent in the latter (see Table S9 for a detail on the parameters used in the calculations).

Table S 9: parameters used for the calculation of the value of $MCI_{solvent}$, considering a solvolysis process such as the one described in this work applied on a 10 g CFRP material and employing a 50%*wt*/50%*wt* mix of recycled and virgin solvent as input.

**Solvent**	
M	33.3
FR	0.5
FU	0
FS	0
V	16.7
CR	1
CU	0
CC	0
CE	0
W0	0
EC	0.9
WC	3.3
EF	0.9
WF	1.85
W	2.59
LFI	0.29
L	1
Lav	1
U	1
Uav	1
X	1
**$MCI_{solvent}$**	**0.74**

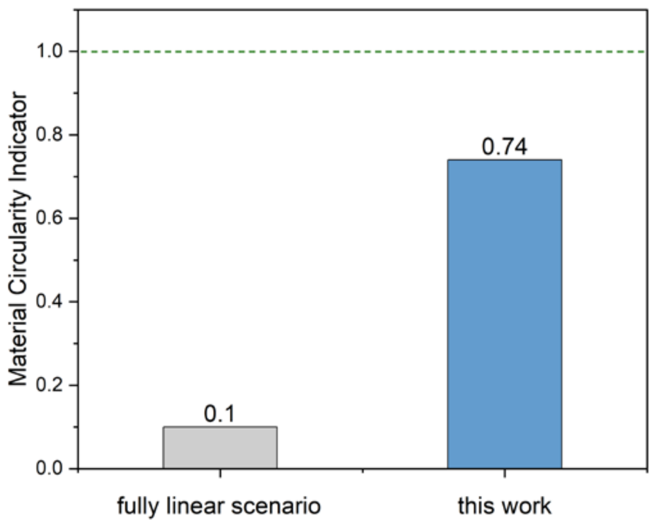

Figure S 34: Comparison of $MCI_{solvent}$ values between base-case scenario (fully linear, only virgin feedstock used) and the scenario characteristic of the process presented in this work.

S13.2 MCI for the modified-catalyst

In line with the previous calculations, the MCI was computed also for the modified-catalyst employed in the reaction, since 90%*wt* of it is constituted by recycled catalyst material recovered from previous solvolysis steps (more information is reported in the main text).

Based on the experimental results reported in this work (see main text), the following considerations can be made. Starting from 10 g of CFRP and a CFRP/solvent mass ratio for the solvolysis reaction of 30%*wt* (thus, 33.3 g of solvent as input), a 5%*wt* concentration of modified-catalyst with respect to the total mass of CFRP and solvent was found as optimized condition for the solvolysis process presented in this work. This yields a mass of solvent of M = 2.17 g.

As demonstrated in this work (see main text), the catalytic mixture is composed of 90%*wt* of organometallic Al-coordination complex (isolated from previous solvolysis reaction and recycled, at a recovery rate of at least 90%, including a conservative 10% of losses occurring during work-up procedures; thus, E_F = 0.9) and 10%*wt* of fresh $AlCl_3$ (hence, F_R = 0.9). Accordingly, $V = M * (1 – F_R – F_U – F_S) = 0.22$ (F_U and F_S are both nil, as this calculation does not consider the presence of reused feedstock or feedstock from biological sources).

At the end of the process, the modified-catalyst can still be recovered by filtration from the organic fraction and subsequently recycled for further processes (C_R = 1, the whole of it will be recyclable), with a recycling efficiency of ~90% (E_C = 0.9), once again considering a conservative 10% of product losses occurring during precipitation and filtration. Based on these considerations, the total amount of waste (W) and the LFI can be computed, yielding values of 0.22 and 0.1, respectively.

Assuming also in this case the utility factor X = 1 (L/L_{av} = 1; U/U_{av} = 1), the calculation of the MCI for the modified-catalyst employed in the solvolysis process described in this work yields $MCI_{catalyst}$ = 0.91. As shown in Figure S35, an evident increase in the level of circularity (*viz*., $MCI_{catalyst}$) for the catalyst is recorded for the scenario characteristic of the process presented in this work with respect to the fully-linear base-case situation (virgin catalyst) (see Table S10 for a detail on the parameters used in the calculations).

Table S 10: parameters used for the calculation of the value of $MCI_{catalyst}$, considering a solvolysis process such as the one described in this work applied on a 10 g CFRP material and 33.3 g of solvent, and employing a catalytic system based on a 90%*wt*/10%*wt* mix of recycled catalyst and virgin catalyst as input.

**Modified-catalyst**	
M	2.17
F_R	0.9
F_U	0
F_S	0
V	0.22
C_R	1
C_U	0
C_C	0
C_E	0
W_0	0
E_C	0.9
W_C	0.22
E_F	0.9
W_F	0.22
W	0.22
LFI	0.1
L	1
L_{av}	1
U	1
U_{av}	1
X	1
**$MCI_{catalyst}$**	**0.91**

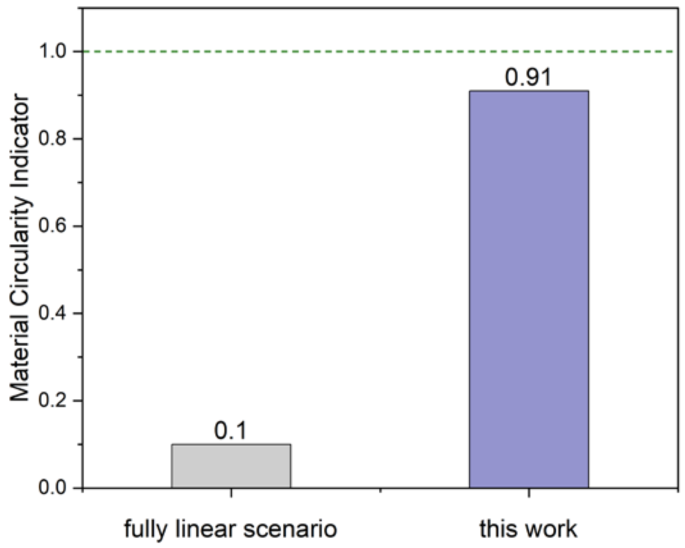

Figure S 35: Comparison of $MCI_{catalyst}$ values between base-case scenario (fully linear, only virgin feedstock used) and the scenario characteristic of the process presented in this work.

S13.3 MCI for the second-generation CFRP

A further assessment of the potential impact of the solvolysis process on the circularity of CFRPs was also carried out by calculating the MCI for second-generation CFRP materials incorporating rCFs reclaimed from the solvolysis process as only reinforcement (100% rCFs), and a fraction (10%*wt*) of the organic recyclate recovered from the chemical recycling process in the resin formulation.

The methodological approach adopted consisted in the computation of the MCI values for the polymeric matrix (resin material) and for the CFs separately, which were then combined to eventually obtain a comprehensive MCI for a representative second-generation CFRP incorporating 60%*wt* of CFs (namely, a total mass of 10 g of CFRP (M_{CFRP} = 10 g), in which M_{CFs} = 6 g and M_{resin} = 4 g).

As mentioned, the polymeric matrix used as resin system for the second-generation CFRP incorporates 90%*wt* of virgin epoxy resin material and 10%*wt* of recycled oligomers, the latter obtained and isolated at the end of the solvolysis process (recovery rate ~90% (E_F = 0.9), considering ~10% of material losses occurring during work-up and vacuum distillation procedures); hence, F_R = 0.1. Consequently, the amount of virgin material (V) constituting the matrix is V = M * (1 – F_R – F_U – F_S) = 3.6 (F_U and F_S are both nil, as this calculation does not consider the presence of reused feedstock or feedstock from biological sources).

Based on these considerations, the total amount of waste (W) and the LFI for the resin can be computed, yielding values for the resin material of 0.22 and 0.49, respectively. Assuming also in this case the utility factor X = 1 (L/L_{av} = 1; U/U_{av} = 1), the calculation of the MCI for the resin material constituting the polymer matrix of the second generation CFRP can be computed (MCI_{resin} = 0.56, see Table S11).

Similarly, the fraction of the MCI for the CF-based reinforcement can be evaluated (MCI_{CFs}), considering the situation of a second-generation CFRP incorporating only rCFs (F_R = 1) reclaimed at the end of a previous solvolysis process with a 90% recycling process efficiency (E_F = 0.9). Also in this case, the CFs can be successively recycled (C_R = 1; with a 90% recycling process efficiency, E_c = 0.9). Because in this scenario only recycled CFs are considered, V = 0 (no virgin fibers are used). Based on these considerations, the total amount of waste (W) and the LFI for the CFs can be computed, yielding values for the CFs 0.63 and 0.05, respectively.

Assuming also in this case the utility factor X = 1 (L/L_{av} = 1; U/U_{av} = 1), the calculation of the MCI for the rCFs incorporated in the second generation CFRP can be computed (MCI_{CFs} = 0.95, see Table S11).

Table S 11: Parameters used for the calculation of the value of MCI_{resin} and MCI_{CFs} in the case of a representative second-generation CFRP incorporating 60%*wt* of CFs; the CFs used for the fabrication of this CFRP are 100% rCFs obtained from a previous solvolysis process; the resin formulation for the polymeric matrix incorporates 90%*wt* of virgin epoxy resin material and 10%*wt* of recycled oligomers isolated at the end of the solvolysis process.

Polymeric matrix		CFs	
M	4	M	6
F_R	0.1	F_R	1
F_U	0	F_U	0
F_S	0	F_S	0
V	3.6	V	0
C_R	1	C_R	1
C_U	0	C_U	0
C_C	0	C_C	0
C_E	0	C_E	0
W_0	0	W_0	0
E_C	0.9	E_C	0.9
W_C	0.4	W_C	0.6
E_F	0.9	E_F	0.9
W_F	0.04	W_F	0.67
W	0.22	W	0.63
LFI	0.49	LFI	0.05
L	1	L	1
L_{av}	1	L_{av}	1
U	1	U	1
U_{av}	1	U_{av}	1
X	1	X	1
**MCI_{resin}**	**0.56**	**MCI_{CFs}**	**0.95**

Finally, the comprehensive MCI for the representative second-generation CFRP (60%*wt* reinforcement) is evaluated as a weight-averaged summation of the previously determined MCI_{resin} and MCI_{CFs}, resulting in MCI_{rCFRP} = 0.80 (Table S12).

As shown in Figure S36, the representative second-generation CFRP fabricated and characterized in this work exhibit a substantially higher MCI (0.8) with respect to a reference CFRP composed only by virgin feedstock, thus confirming the positive impact the solvolysis process developed in this work can have on the circularity of future composite systems.

Table S 12: Parameters used for the calculation of the value of the MCI for a representative second-generation CFRP incorporating 60%*wt* of CFs; the CFs used for the fabrication of this CFRP are 100% rCFs obtained from a previous solvolysis process; the resin formulation for the polymeric matrix incorporates 90%*wt* of virgin epoxy resin material and 10%*wt* of recycled oligomers isolated at the end of the solvolysis process; MCI_{rCFRP} is calculated as weight-averaged summation of MCI_{resin} and MCI_{CFs}.

Second-gen. CFRP	
V	3.6
W	0.9
M	10
LFI	0.2
X	1.0
**MCI_{rCFRP}**	**0.80**

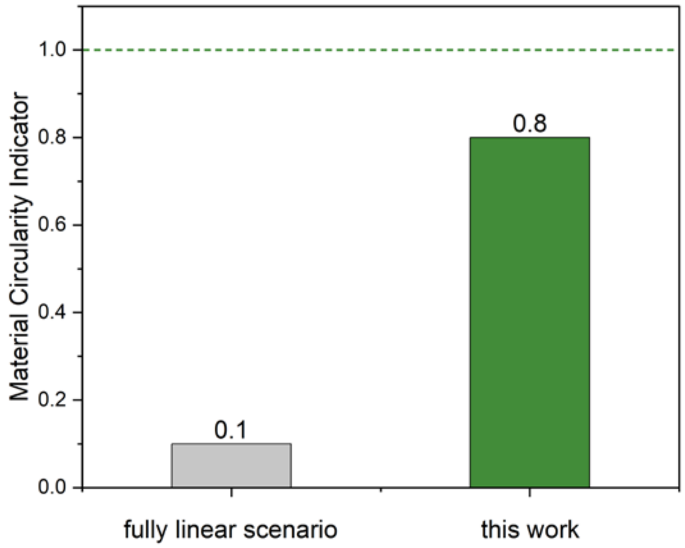

Figure S 36: Comparison of MCI values for the second-generation CFRP between base-case scenario (CFRP incorporating virgin CFs and virgin resin) and the scenario characteristic of this work (CFRP incorporating only rCFs as reinforcement and a resin formulation based on 90%*wt* of virgin epoxy resin and 10%*wt* recycled oligomeric fraction recovered from the chemical recycling process).

Bibliography

S1. European Chemical Agency - ECHA.

S2. C. M. Hansen, *Hansen Solubility Parameters: A User's Handbook.* (CRC press, 2007).

S3. D. W. Van Krevelen, K. Te Nijenhuis, "Polymer Properties" in *Properties of Polymers* (Elsevier, 2009), pp. 3–5.

S4. H. Launay, C. M. Hansen, K. Almdal, Hansen solubility parameters for a carbon fiber/epoxy composite. *Carbon N Y* 45, 2859–2865 (2007).

S5. A. Beaugendre, S. Saidi, S. Degoutin, S. Bellayer, C. Pierlot, S. Duquesne, M. Casetta, M. Jimenez, One pot flame retardant and weathering resistant coatings for plastics: A novel approach. *RSC Adv* 7, 40682–40694 (2017).

S6. G. Altamura, E. Manarin, G. Griffini, S. Turri, Revisiting Unsaturated Polyesters: Recyclable-by-Design Vinylester Resins for the Circular Economy. *Eur Polym J* 228, 113819 (2025).
